# Supplementary material for: Dengue transmission dynamics in an urban setting in western India
Source: PLoS Negl Trop Dis. 2026 Mar 23;20(3):e0013636. doi: 10.1371/journal.pntd.0013636 (PMC13052988; doi:10.1371/journal.pntd.0013636)
Supplement: S1 Fig — (DOCX) [file pntd.0013636.s001.docx]

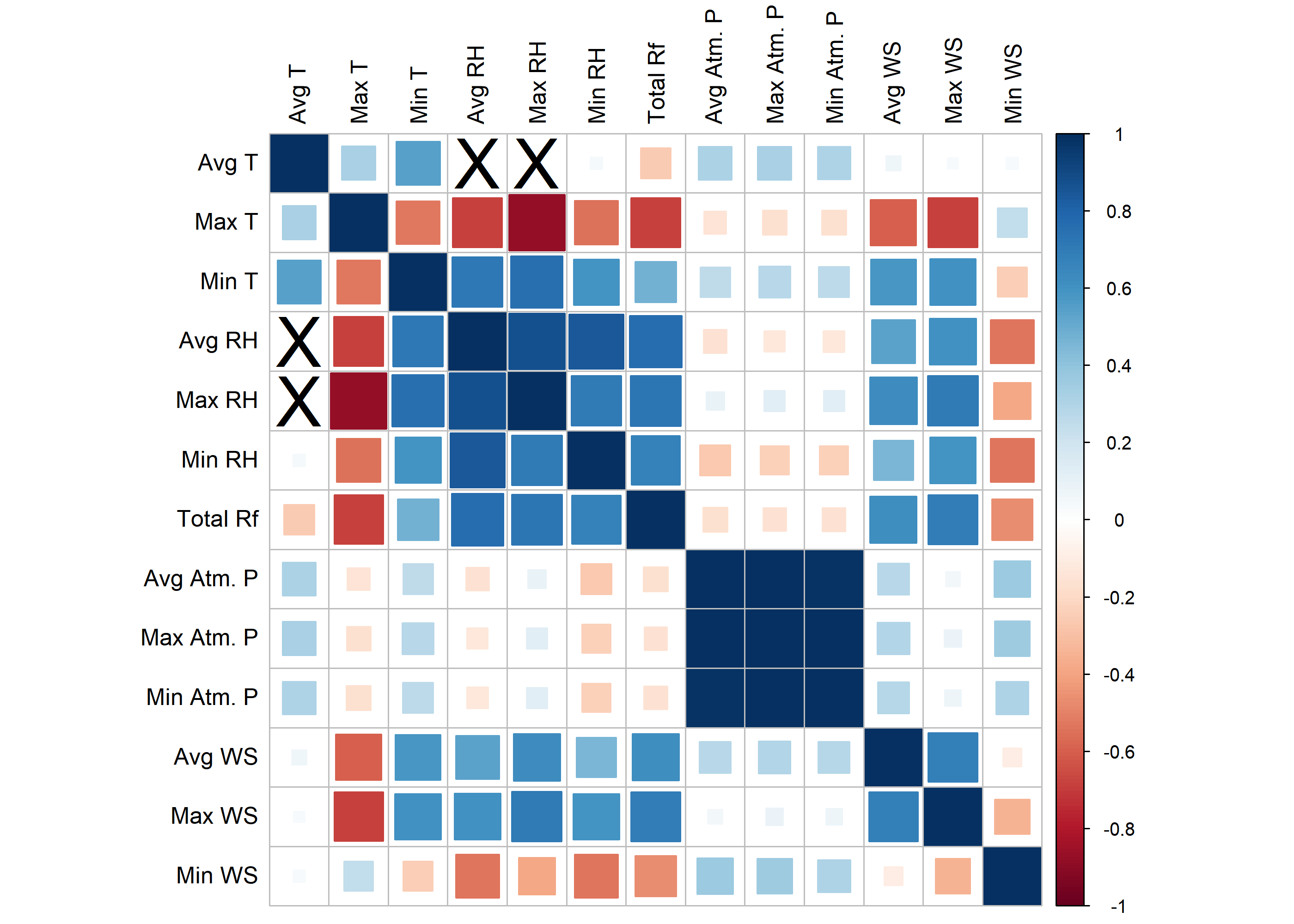


**S1 Fig:** Multicollinearity between 13 climatic variables based on Pearson’s correlation factor. The colour of each square indicates the Pearson’s correlation value, whereas the size of the square indicates the overall significance of the correlation. Squares marked as X did not have any significant effect.
