# Supplementary material for: Dengue transmission dynamics in an urban setting in western India
Source: PLoS Negl Trop Dis. 2026 Mar 23;20(3):e0013636. doi: 10.1371/journal.pntd.0013636 (PMC13052988; doi:10.1371/journal.pntd.0013636)
Supplement: S2 Fig — (DOCX) [file pntd.0013636.s002.docx]

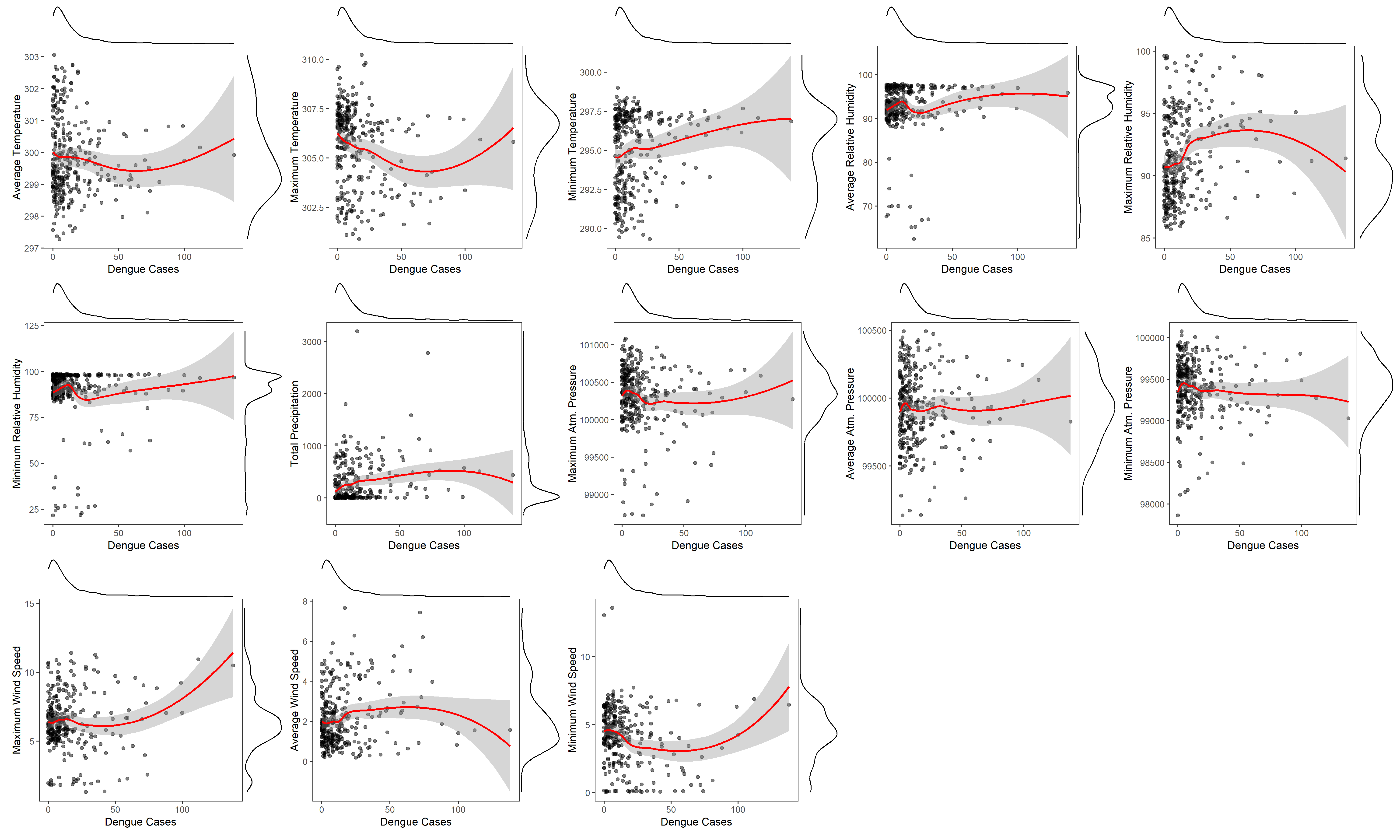


**S2 Fig:** Scatter plots of the 13 climatic predictors against dengue incidence in all 34 PHC/UHCs in Goa to assess the patterns of relationship between the predictors and dengue incidence. As cases were mostly few, most of the points are concentred in the lower left part of the graphs and only limited information is available regarding the impact relationship structure at high dengue incidence rates.
