# Supplementary material for: Dengue transmission dynamics in an urban setting in western India
Source: PLoS Negl Trop Dis. 2026 Mar 23;20(3):e0013636. doi: 10.1371/journal.pntd.0013636 (PMC13052988; doi:10.1371/journal.pntd.0013636)
Supplement: S3 Fig — (DOCX) [file pntd.0013636.s003.docx]

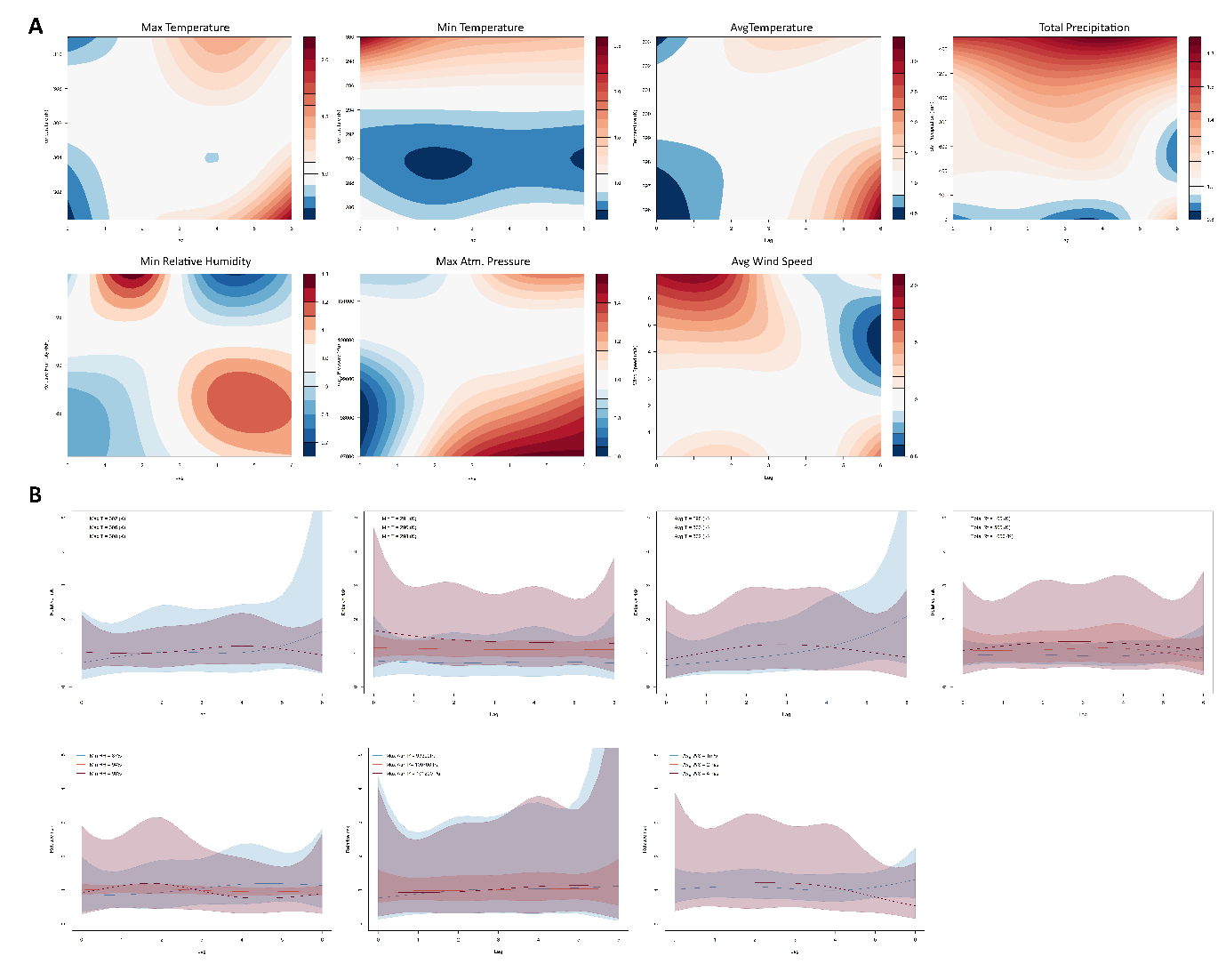


**S3 Fig**: (A) Contour plots to show the exposure-lag-response of the single variable models for each of the seven most significant climatic predictors for dengue and (B) Lag dependent relative risk curve of dengue for each of these variables at the 10^th^, 50^th^ and 90^th^ percentiles
