# Supplementary material for: Dengue transmission dynamics in an urban setting in western India
Source: PLoS Negl Trop Dis. 2026 Mar 23;20(3):e0013636. doi: 10.1371/journal.pntd.0013636 (PMC13052988; doi:10.1371/journal.pntd.0013636)
Supplement: S1 Table — (DOCX) [file pntd.0013636.s004.docx]

**S1** **Table:** Dengue case data from 2011 to 2024 and annual average incidence in the state of Goa

| **PHC/UHC/CHC** | **Estimated Population** | **2011** | **2012** | **2013** | **2014** | **2015** | **2016** | **2017** | **2018** | **2019** | **2020** | **2021** | **2022** | **2023** | **2024** | **Total Cases** | **Average annual Incidence (95%CI)** |  |
| --- | --- | --- | --- | --- | --- | --- | --- | --- | --- | --- | --- | --- | --- | --- | --- | --- | --- | --- |
| **Panaji** | 29865 | 1 | 6 | 7 | 8 | 2 | 3 | 17 | 18 | 7 | 39 | 58 | 13 | 14 | 4 | 197 | 33.7 (28.4–39.8) |  |
| **Mapusa** | 35172 | 1 | 0 | 10 | 10 | 7 | 5 | 3 | 15 | 72 | 18 | 55 | 67 | 100 | 74 | 437 | 4.7 (3–7) |  |
| **Pernem** | 43391 | 0 | 0 | 2 | 1 | 2 | 6 | 4 | 2 | 30 | 5 | 13 | 27 | 27 | 27 | 146 | 16.5 (13.4–20) |  |
| **Candolim** | 38862 | 0 | 1 | 15 | 47 | 4 | 3 | 30 | 18 | 114 | 136 | 37 | 57 | 38 | 21 | 521 | 95.8 (87.7–104.3) |  |
| **Aldona** | 24854 | 1 | 1 | 12 | 10 | 5 | 8 | 3 | 3 | 21 | 4 | 16 | 26 | 16 | 15 | 141 | 16.4 (12.4–21.2) |  |
| **Bicholim** | 38897 | 0 | 0 | 1 | 1 | 2 | 5 | 3 | 1 | 19 | 6 | 10 | 14 | 18 | 20 | 100 | 38.4 (33.4–44) |  |
| **Valpoi** | 48007 | 1 | 15 | 4 | 3 | 10 | 14 | 11 | 23 | 15 | 6 | 7 | 9 | 5 | 12 | 135 | 8.6 (6.6–11.2) |  |
| **Betqui** | 83723 | 0 | 1 | 2 | 3 | 2 | 1 | 7 | 5 | 1 | 0 | 0 | 0 | 0 | 1 | 23 | 5.3 (4.1–6.8) |  |
| **Cansarvanem** | 44875 | 1 | 0 | 2 | 1 | 0 | 1 | 2 | 0 | 5 | 2 | 11 | 10 | 13 | 9 | 57 | 69.6 (63.2–76.4) |  |
| **Siolim** | 40017 | 1 | 1 | 0 | 1 | 1 | 4 | 4 | 5 | 22 | 3 | 25 | 29 | 31 | 56 | 183 | 35.2 (30.4–40.4) |  |
| **Colvale** | 55412 | 0 | 0 | 3 | 1 | 2 | 2 | 2 | 10 | 28 | 5 | 39 | 37 | 37 | 43 | 209 | 18.8 (15.9–22.1) |  |
| **Corlim** | 63313 | 2 | 3 | 10 | 16 | 3 | 8 | 7 | 2 | 2 | 0 | 1 | 0 | 0 | 4 | 58 | 12.3 (10.1–14.8) |  |
| **Sanquelim** | 46434 | 1 | 0 | 0 | 0 | 0 | 2 | 2 | 1 | 9 | 18 | 5 | 8 | 38 | 25 | 109 | 28.2 (24.2–32.5) |  |
| **Porvorim** | 44545 | 0 | 0 | 0 | 0 | 0 | 0 | 19 | 17 | 21 | 17 | 22 | 10 | 17 | 19 | 142 | 21.6 (18.1–25.6) |  |
| **Mayem** | 38458 | 0 | 0 | 0 | 0 | 0 | 0 | 1 | 2 | 2 | 1 | 7 | 5 | 8 | 5 | 31 | 26.4 (22.2–31.1) |  |
| **Chimbel** | 26032 | 0 | 0 | 0 | 0 | 0 | 0 | 0 | 4 | 5 | 9 | 14 | 6 | 18 | 6 | 62 | 8.5 (5.8–12.1) |  |
| **Saligao** | 23617 | 0 | 0 | 0 | 0 | 0 | 0 | 0 | 0 | 0 | 0 | 0 | 18 | 42 | 62 | 122 | 36.9 (30.6–44.1) |  |
| **North Goa TOTAL** | **725474** | **9** | **28** | **68** | **102** | **40** | **62** | **115** | **126** | **373** | **269** | **320** | **336** | **422** | **403** | **2673** |  |  |
| **Margao** | 44985 | 2 | 1 | 10 | 2 | 32 | 16 | 7 | 27 | 37 | 4 | 3 | 31 | 23 | 46 | 241 | 12.5 (9.9–15.6) |  |
| **Vasco** | 49221 | 4 | 4 | 18 | 4 | 64 | 11 | 43 | 41 | 33 | 60 | 227 | 19 | 25 | 10 | 563 | 30.8 (26.8–35.2) |  |
| **Ponda** | 50285 | 1 | 0 | 22 | 1 | 3 | 3 | 2 | 16 | 3 | 2 | 0 | 0 | 8 | 4 | 65 | 7.4 (5.5–9.7) |  |
| **Marcaim** | 34389 | 0 | 1 | 12 | 2 | 0 | 2 | 3 | 49 | 2 | 1 | 0 | 0 | 0 | 0 | 72 | 11.2 (8.4–14.6) |  |
| **Shiroda** | 68876 | 3 | 2 | 6 | 7 | 22 | 11 | 5 | 14 | 35 | 2 | 1 | 0 | 1 | 3 | 112 | 27.9 (24.7–31.4) |  |
| **Cansaulim** | 48585 | 0 | 0 | 6 | 8 | 6 | 6 | 2 | 2 | 5 | 3 | 0 | 0 | 2 | 12 | 52 | 4.1 (2.7–5.9) |  |
| **Curtorim** | 53965 | 1 | 0 | 10 | 5 | 66 | 5 | 2 | 6 | 16 | 3 | 0 | 6 | 2 | 14 | 136 | 18 (15.1–21.3) |  |
| **Bali** | 35520 | 0 | 0 | 13 | 8 | 9 | 5 | 3 | 3 | 10 | 1 | 2 | 9 | 0 | 16 | 79 | 13.1 (10.1–16.7) |  |
| **Canacona** | 98858 | 3 | 0 | 7 | 7 | 6 | 2 | 9 | 7 | 133 | 1 | 0 | 11 | 7 | 19 | 212 | 17.4 (15.3–19.8) |  |
| **Curchorem** | 32507 | 0 | 1 | 3 | 2 | 2 | 2 | 3 | 4 | 4 | 2 | 0 | 4 | 0 | 1 | 28 | 3.7 (2.2–6) |  |
| **Sanguem** | 38794 | 0 | 0 | 1 | 0 | 1 | 0 | 1 | 0 | 2 | 0 | 0 | 0 | 1 | 0 | 6 | 7.2 (5.1–9.8) |  |
| **Cortalim** | 25138 | 1 | 0 | 6 | 11 | 19 | 8 | 16 | 12 | 35 | 23 | 94 | 21 | 11 | 12 | 269 | 1.7 (0.6–3.7) |  |
| **Loutolim** | 97321 | 0 | 1 | 7 | 4 | 9 | 10 | 13 | 4 | 3 | 0 | 0 | 1 | 4 | 9 | 65 | 41.3 (38–44.9) |  |
| **Chinchinim** | 25862 | 2 | 1 | 9 | 4 | 7 | 2 | 6 | 7 | 12 | 0 | 0 | 1 | 1 | 2 | 54 | 19.9 (15.6–25) |  |
| **Quepem** | 28918 | 0 | 0 | 0 | 0 | 6 | 2 | 3 | 4 | 5 | 3 | 1 | 2 | 3 | 10 | 39 | 27.7 (22.8–33.3) |  |
| **Dharbandora** | 77548 | 0 | 0 | 0 | 1 | 1 | 3 | 2 | 4 | 3 | 2 | 0 | 0 | 0 | 1 | 17 | 6 (4.6–7.6) |  |
| **Navelim** | 35349 | 0 | 0 | 0 | 0 | 0 | 0 | 0 | 9 | 15 | 0 | 1 | 2 | 2 | 5 | 34 | 6.9 (4.8–9.6) |  |
| **South Goa Total** | **846121** | **17** | **11** | **130** | **66** | **253** | **88** | **120** | **209** | **353** | **107** | **329** | **107** | **90** | **164** | **2044** |  |  |
| **TOTAL CASES** | **1571595** | **26** | **39** | **198** | **168** | **293** | **150** | **235** | **335** | **726** | **376** | **649** | **443** | **512** | **567** | **4717** |  |  |
| **Incidence (95%CI) per 100,000 person years** |  | 1.8 (1.2–2.6) | 2.7 (1.9–3.7) | 13.7 (11.9–15.8) | 11.6 (9.9–13.5) | 20.1 (17.9–22.5) | 10.3 (8.7–12.1) | 16.1 (14.1–18.3) | 23 (20.7–25.7) | 49.9 (46.3–53.6) | 25.8 (23.3–28.5) | 44.5 (41.2–48.1) | 30.5 (27.7–33.4) | 35.3 (32.3–38.5) | 39.1 (36–42.4) | **23.1 (22.5–23.8)** |  |  |
|  |  |  |  |  |  |  |  |  |  |  |  |  |  |  |  |  |  |  |
| **DEATHS** |  | 0 | 0 | 2 | 1 | 0 | 0 | 0 | 1 | 1 | 0 | 0 | 1 | 3 | 3 | 12 |  |  |
