# Supplementary material for: Dengue transmission dynamics in an urban setting in western India
Source: PLoS Negl Trop Dis. 2026 Mar 23;20(3):e0013636. doi: 10.1371/journal.pntd.0013636 (PMC13052988; doi:10.1371/journal.pntd.0013636)
Supplement: S2 Table — (DOCX) [file pntd.0013636.s005.docx]

**S2 Table:** Metadata of satellite datasets used in the study

| **Satellite** | **Landsat 5** | **Landsat 9** |
| --- | --- | --- |
| **Sensor ID** | LANDSAT/LT05/C02/T1_L2 | LANDSAT/LC09/C02/T1_L2 |
| **Date of acquisition** | Feb 01 - Feb 28 1991 | Feb 01 - Feb 29 2024 |
| **Columns and Rows** | 3740, 4639 | 3740, 4639 |
| **Cell Size(X,Y)** | 30,30 | 30,30 |
| **Coordinate System** | WGS_1985_UTM_Zone_45N | WGS_1985_UTM_Zone_45N |
| **Linear Unit** | Meter | Meter |
| **Central Meridian** | 87 | 87 |
| **Scale Factor** | 0.9996 | 0.9996 |
| **No. of Bands** | 1,2,3,4,5,7 | 1,2,3,4,5,6 |
| **Cloud Cover** | <10% | <10% |
| **EPSG Code** | 32645 | 32645 |
