# Supplementary material for: Dengue transmission dynamics in an urban setting in western India
Source: PLoS Negl Trop Dis. 2026 Mar 23;20(3):e0013636. doi: 10.1371/journal.pntd.0013636 (PMC13052988; doi:10.1371/journal.pntd.0013636)
Supplement: S3 Table — (DOCX) [file pntd.0013636.s006.docx]

**S3 Table:** Land use / Land cover (LU/LC) area composition between 1991 and 2024

| **Class Name** | **Area in 1991 (sq.km)** | **Area in 2024 (sq.km)** |
| --- | --- | --- |
| **Agriculture** | 175.93 | 107.73 |
| **Built-up** | 67.93 | 258.64 |
| **Forest** | 2644.73 | 2596.58 |
| **Mining** | 0.67 | 4.67 |
| **Misc** | 83.89 | 236.46 |
| **Scrubland** | 669.19 | 449.93 |
| **Water** | 161.95 | 179.36 |
| **Wetland** | 73.74 | 44.65 |
