# Supplementary material for: Dengue transmission dynamics in an urban setting in western India
Source: PLoS Negl Trop Dis. 2026 Mar 23;20(3):e0013636. doi: 10.1371/journal.pntd.0013636 (PMC13052988; doi:10.1371/journal.pntd.0013636)
Supplement: S4 Table — (DOCX) [file pntd.0013636.s007.docx]

S4 Table: Summary of Accuracy across Locations and of Time Series Outliers

| Summary of Accuracy across Locations | |  |  |  |  |
| --- | --- | --- | --- | --- | --- |
| **Category** | **Min** | **Max** | **Mean** | **Median** | **Std. Dev.** |
| Forecast RMSE | 0.38 | 40.83 | 7.79 | 4.68 | 8.8 |
| Validation RMSE | 0.06 | 43.55 | 11.93 | 6.69 | 12.59 |

| Summary of Time Series Outliers | |
| --- | --- |
| Number of locations containing outliers | 11 |
| Percent of locations containing outliers | 32.35 |
| Number of outliers by location (Min; Mean; Max) | 0; 0.32; 1 |
| Number of outliers by time step (Min; Mean; Max) | 0; 0.92; 3 |
| Time step containing largest number of outliers | on or after |
|  | 01-01-2018 00:00 |
|  | to before |
|  | 01-01-2019 00:00 |

| SN | PHC/UHC | 2011 | 2012 | 2013 | 2014 | 2015 | 2016 | 2017 | 2018 | 2019 | 2020 | 2021 | 2022 | 2023 | 2024 |
| --- | --- | --- | --- | --- | --- | --- | --- | --- | --- | --- | --- | --- | --- | --- | --- |
| 1 | Aldona | 1 | 1 | 12 | 10 | 5 | 8 | 3 | 3 | 21 | 4 | 16 | 26 | 16 | 15 |
| 2 | Bali | 0 | 0 | 13 | 8 | 9 | 5 | 3 | 3 | 10 | 1 | 2 | 9 | 0 | 16 |
| 3 | Betqui | 0 | 1 | 2 | 3 | 2 | 1 | 7 | 5 | 1 | 0 | 0 | 0 | 0 | 1 |
| 4 | Bicholim | 0 | 0 | 1 | 1 | 2 | 5 | 3 | 1 | 19 | 6 | 10 | 14 | 18 | 20 |
| 5 | Canacona | 3 | 0 | 7 | 7 | 6 | 2 | 9 | 7 | 133 | 1 | 0 | 11 | 7 | 19 |
| 6 | Candolim | 0 | 1 | 15 | 47 | 4 | 3 | 30 | 18 | 114 | 136 | 37 | 57 | 38 | 21 |
| 7 | Cansarvanem | 1 | 0 | 2 | 1 | 0 | 1 | 2 | 0 | 5 | 2 | 11 | 10 | 13 | 9 |
| 8 | Cansaulim | 0 | 0 | 6 | 8 | 6 | 6 | 2 | 2 | 5 | 3 | 0 | 0 | 2 | 12 |
| 9 | Chimbel | 0 | 0 | 0 | 0 | 0 | 0 | 0 | 4 | 5 | 9 | 14 | 6 | 18 | 6 |
| 10 | Chinchinim | 2 | 1 | 9 | 4 | 7 | 2 | 6 | 7 | 12 | 0 | 0 | 1 | 1 | 2 |
| 11 | Colvale | 0 | 0 | 3 | 1 | 2 | 2 | 2 | 10 | 28 | 5 | 39 | 37 | 37 | 43 |
| 12 | Corlim | 2 | 3 | 10 | 16 | 3 | 8 | 7 | 2 | 2 | 0 | 1 | 0 | 0 | 4 |
| 13 | Cortalim | 1 | 0 | 6 | 11 | 19 | 8 | 16 | 12 | 35 | 23 | 94 | 21 | 11 | 12 |
| 14 | Curchorem | 0 | 1 | 3 | 2 | 2 | 2 | 3 | 4 | 4 | 2 | 0 | 4 | 0 | 1 |
| 15 | Curtorim | 1 | 0 | 10 | 5 | 66 | 5 | 2 | 6 | 16 | 3 | 0 | 6 | 2 | 14 |
| 16 | Dharbandora | 0 | 0 | 0 | 1 | 1 | 3 | 2 | 4 | 3 | 2 | 0 | 0 | 0 | 1 |
| 17 | Loutolim | 0 | 1 | 7 | 4 | 9 | 10 | 13 | 4 | 3 | 0 | 0 | 1 | 4 | 9 |
| 18 | Mapusa | 1 | 0 | 10 | 10 | 7 | 5 | 3 | 15 | 72 | 18 | 55 | 67 | 100 | 74 |
| 19 | Marcaim | 0 | 1 | 12 | 2 | 0 | 2 | 3 | 49 | 2 | 1 | 0 | 0 | 0 | 0 |
| 20 | Margao | 2 | 1 | 10 | 2 | 32 | 16 | 7 | 27 | 37 | 4 | 3 | 31 | 23 | 46 |
| 21 | Mayem | 0 | 0 | 0 | 0 | 0 | 0 | 1 | 2 | 2 | 1 | 7 | 5 | 8 | 5 |
| 22 | Navelim | 0 | 0 | 0 | 0 | 0 | 0 | 0 | 9 | 15 | 0 | 1 | 2 | 2 | 5 |
| 23 | Panaji | 1 | 6 | 7 | 8 | 2 | 3 | 17 | 18 | 7 | 39 | 58 | 13 | 14 | 4 |
| 24 | Pernem | 0 | 0 | 2 | 1 | 2 | 6 | 4 | 2 | 30 | 5 | 13 | 27 | 27 | 27 |
| 25 | Ponda | 1 | 0 | 22 | 1 | 3 | 3 | 2 | 16 | 3 | 2 | 0 | 0 | 8 | 4 |
| 26 | Porvorim | 0 | 0 | 0 | 0 | 0 | 0 | 19 | 17 | 21 | 17 | 22 | 10 | 17 | 19 |
| 27 | Quepem | 0 | 0 | 0 | 0 | 6 | 2 | 3 | 4 | 5 | 3 | 1 | 2 | 3 | 10 |
| 28 | Saligao | 0 | 0 | 0 | 0 | 0 | 0 | 0 | 0 | 0 | 0 | 0 | 18 | 42 | 62 |
| 29 | Sanguem | 0 | 0 | 1 | 0 | 1 | 0 | 1 | 0 | 2 | 0 | 0 | 0 | 1 | 0 |
| 30 | Sanquelim | 1 | 0 | 0 | 0 | 0 | 2 | 2 | 1 | 9 | 18 | 5 | 8 | 38 | 25 |
| 31 | Shiroda | 3 | 2 | 6 | 7 | 22 | 11 | 5 | 14 | 35 | 2 | 1 | 0 | 1 | 3 |
| 32 | Siolim | 1 | 1 | 0 | 1 | 1 | 4 | 4 | 5 | 22 | 3 | 25 | 29 | 31 | 56 |
| 33 | Valpoi | 1 | 15 | 4 | 3 | 10 | 14 | 11 | 23 | 15 | 6 | 7 | 9 | 5 | 12 |
| 34 | Vasco | 4 | 4 | 18 | 4 | 64 | 11 | 43 | 41 | 33 | 60 | 227 | 19 | 25 | 10 |

| SN | PHC/UHC | Forecast 2025 | Forecast 2026 | Forecast 2027 | Forecast 2028 | Forecast 2029 | Forecast RMSE | V RMSE | TIME WINDOW | IS SEASON | N  OUTLIERS |
| --- | --- | --- | --- | --- | --- | --- | --- | --- | --- | --- | --- |
| 1 | Aldona | 14 | 14 | 14 | 14 | 14 | 5 | 1 | 2 | 1 | 0 |
| 2 | Bali | 4 | 6 | 5 | 5 | 5 | 3 | 11 | 2 | 1 | 0 |
| 3 | Betqui | 2 | 2 | 2 | 3 | 3 | 1 | 1 | 3 | 0 | 0 |
| 4 | Bicholim | 16 | 16 | 16 | 16 | 16 | 4 | 7 | 2 | 1 | 0 |
| 5 | Canacona | 41 | 41 | 41 | 41 | 41 | 27 | 23 | 2 | 1 | 1 |
| 6 | Candolim | 39 | 41 | 38 | 38 | 40 | 29 | 28 | 3 | 0 | 0 |
| 7 | Cansarvanem | 11 | 11 | 11 | 11 | 11 | 2 | 2 | 2 | 1 | 0 |
| 8 | Cansaulim | 3 | 3 | 3 | 2 | 3 | 2 | 10 | 3 | 0 | 0 |
| 9 | Chimbel | 13 | 9 | 13 | 9 | 13 | 3 | 5 | 2 | 1 | 0 |
| 10 | Chinchinim | 5 | 6 | 7 | 5 | 5 | 2 | 4 | 3 | 0 | 0 |
| 11 | Colvale | 38 | 38 | 38 | 38 | 38 | 7 | 9 | 2 | 1 | 1 |
| 12 | Corlim | 2 | 2 | 2 | 2 | 2 | 3 | 3 | 3 | 0 | 1 |
| 13 | Cortalim | 30 | 30 | 50 | 42 | 42 | 16 | 43 | 3 | 0 | 1 |
| 14 | Curchorem | 3 | 4 | 3 | 4 | 3 | 3 | 17 | 2 | 1 | 1 |
| 15 | Curtorim | 21 | 20 | 18 | 18 | 18 | 14 | 4 | 2 | 1 | 1 |
| 16 | Dharbandora | 2 | 2 | 2 | 2 | 2 | 1 | 1 | 3 | 0 | 0 |
| 17 | Loutolim | 6 | 7 | 8 | 8 | 8 | 2 | 6 | 3 | 0 | 0 |
| 18 | Mapusa | 72 | 68 | 72 | 68 | 72 | 14 | 8 | 2 | 1 | 0 |
| 19 | Marcaim | 6 | 17 | 13 | 12 | 12 | 11 | 20 | 2 | 1 | 1 |
| 20 | Margao | 25 | 29 | 25 | 29 | 25 | 9 | 34 | 2 | 1 | 0 |
| 21 | Mayem | 6 | 6 | 6 | 6 | 6 | 1 | 1 | 3 | 0 | 0 |
| 22 | Navelim | 3 | 3 | 3 | 3 | 3 | 3 | 4 | 3 | 0 | 0 |
| 23 | Panaji | 19 | 16 | 19 | 17 | 19 | 11 | 25 | 3 | 0 | 0 |
| 24 | Pernem | 23 | 23 | 23 | 23 | 23 | 6 | 6 | 2 | 1 | 0 |
| 25 | Ponda | 4 | 4 | 4 | 4 | 4 | 4 | 0 | 3 | 0 | 1 |
| 26 | Porvorim | 19 | 17 | 19 | 17 | 19 | 6 | 2 | 3 | 0 | 0 |
| 27 | Quepem | 3 | 3 | 4 | 4 | 4 | 2 | 7 | 3 | 0 | 0 |
| 28 | Saligao | 38 | 38 | 38 | 38 | 38 | 9 | 44 | 3 | 0 | 0 |
| 29 | Sanguem | 1 | 0 | 1 | 0 | 1 | 0 | 0 | 3 | 0 | 0 |
| 30 | Sanquelim | 18 | 22 | 22 | 22 | 22 | 6 | 9 | 3 | 0 | 1 |
| 31 | Shiroda | 11 | 14 | 14 | 15 | 14 | 7 | 9 | 3 | 0 | 0 |
| 32 | Siolim | 40 | 40 | 40 | 40 | 40 | 7 | 31 | 3 | 0 | 0 |
| 33 | Valpoi | 13 | 14 | 15 | 13 | 11 | 4 | 1 | 3 | 0 | 1 |
| 34 | Vasco | 52 | 109 | 74 | 75 | 75 | 41 | 31 | 2 | 1 | 1 |
