# Supplementary material for: Dengue transmission dynamics in an urban setting in western India
Source: PLoS Negl Trop Dis. 2026 Mar 23;20(3):e0013636. doi: 10.1371/journal.pntd.0013636 (PMC13052988; doi:10.1371/journal.pntd.0013636)
Supplement: S5 Table — (DOCX) [file pntd.0013636.s008.docx]

**S5 Table:** Results of the Generalized Linear Model Regression

| Dep. Variable: | Cases | No. Observations: | 476 |
| --- | --- | --- | --- |
| Model: | GLM | Df Residuals: | 441 |
| Model Family: | Negative Binomial | Df Model: | 34 |
| Link Function: | Log | Scale: | 1 |
| Method: | IRLS | Log-Likelihood | -1376.1 |
| No. Iterations: | 16 | Deviance: | 621.69 |
| Covariance Type: | nonrobust | Pearson chi2: | 616 |
|  |  | Pseudo R-square. (CS) : | 0.5946 |

| **Variable** | **coef** | **std_err** | **z** | **P>\|z\|** | **[0.025]** | **[0.975]** |
| --- | --- | --- | --- | --- | --- | --- |
| const | -382.472 | 26.061 | -14.676 | 0 | -433.55 | -331.394 |
| Year | 0.1907 | 0.013 | 14.764 | 0 | 0.165 | 0.216 |
| Bali | -0.4228 | 0.409 | -1.033 | 0.301 | -1.225 | 0.379 |
| **Betki** | -1.3683 | 0.433 | -3.162 | 0.002 | -2.217 | -0.52 |
| Bicholim | -0.6719 | 0.414 | -1.624 | 0.104 | -1.483 | 0.139 |
| Canacona | 0.3789 | 0.399 | 0.949 | 0.343 | -0.404 | 1.162 |
| **Candolim** | 1.216 | 0.395 | 3.081 | 0.002 | 0.442 | 1.99 |
| **Cansarvanem** | -1.2431 | 0.429 | -2.901 | 0.004 | -2.083 | -0.403 |
| **Cansaulim** | -0.8435 | 0.417 | -2.02 | 0.043 | -1.662 | -0.025 |
| **Chimbel** | -1.2882 | 0.43 | -2.996 | 0.003 | -2.131 | -0.445 |
| Chinchinim | -0.582 | 0.412 | -1.413 | 0.158 | -1.389 | 0.225 |
| Colvale | -0.0652 | 0.404 | -0.161 | 0.872 | -0.857 | 0.727 |
| Corlim | -0.3391 | 0.408 | -0.832 | 0.406 | -1.138 | 0.46 |
| Cortalim | 0.5245 | 0.398 | 1.317 | 0.188 | -0.256 | 1.305 |
| **Curchorem** | -1.3246 | 0.431 | -3.072 | 0.002 | -2.17 | -0.479 |
| Curtorim | 0.3507 | 0.4 | 0.877 | 0.38 | -0.433 | 1.134 |
| Dharbandora | -1.7721 | 0.45 | -3.942 | 0 | -2.653 | -0.891 |
| Loutolim | -0.4344 | 0.409 | -1.062 | 0.288 | -1.237 | 0.368 |
| **Mapusa** | 0.6761 | 0.397 | 1.702 | 0.089 | -0.103 | 1.455 |
| Marcaim | -0.3717 | 0.408 | -0.91 | 0.363 | -1.172 | 0.428 |
| Margao | 0.5466 | 0.398 | 1.373 | 0.17 | -0.234 | 1.327 |
| Mayem | -1.7296 | 0.448 | -3.864 | 0 | -2.607 | -0.852 |
| **Navelim** | -1.5064 | 0.438 | -3.44 | 0.001 | -2.365 | -0.648 |
| Panaji | 0.2979 | 0.4 | 0.745 | 0.457 | -0.486 | 1.082 |
| Pernem | -0.4286 | 0.409 | -1.048 | 0.295 | -1.231 | 0.373 |
| Ponda | -0.2507 | 0.406 | -0.617 | 0.537 | -1.047 | 0.546 |
| Porvorim | -0.3372 | 0.408 | -0.827 | 0.408 | -1.136 | 0.462 |
| **Quepem** | -0.9709 | 0.421 | -2.308 | 0.021 | -1.795 | -0.147 |
| **Saligao** | -1.371 | 0.433 | -3.167 | 0.002 | -2.219 | -0.523 |
| **Sanguem** | -1.4262 | 0.435 | -3.28 | 0.001 | -2.278 | -0.574 |
| **Sanquelim** | -0.827 | 0.417 | -1.983 | 0.047 | -1.644 | -0.01 |
| Shiroda | 0.0944 | 0.402 | 0.235 | 0.814 | -0.694 | 0.883 |
| Siolim | -0.411 | 0.409 | -1.005 | 0.315 | -1.212 | 0.39 |
| Valpoi | 0.1838 | 0.401 | 0.458 | 0.647 | -0.603 | 0.97 |
| **Vasco** | 1.3277 | 0.394 | 3.368 | 0.001 | 0.555 | 2.101 |
