# Supplementary material for: Dengue transmission dynamics in an urban setting in western India
Source: PLoS Negl Trop Dis. 2026 Mar 23;20(3):e0013636. doi: 10.1371/journal.pntd.0013636 (PMC13052988; doi:10.1371/journal.pntd.0013636)
Supplement: S6 Table — (DOCX) [file pntd.0013636.s009.docx]

**S6 Table**: Age and sex wise distribution of dengue cases in North and South Goa districts

|  | **Below 15 Years** | | | **15 years and above** | | | **Total** | | |
| --- | --- | --- | --- | --- | --- | --- | --- | --- | --- |
|  | Male | Female | Total | Male | Female | Total | Male | Female | Total |
| North Goa | 147 | 104 | 251 | 771 | 461 | 1232 | 918 | 565 | 1483 |
| South Goa | 106 | 83 | 189 | 311 | 188 | 499 | 417 | 271 | 688 |
| **Total** | **253** | **187** | **440** | **1082** | **649** | **1731** | **1335** | **836** | **2171** |
