# Supplementary material for: Dengue transmission dynamics in an urban setting in western India
Source: PLoS Negl Trop Dis. 2026 Mar 23;20(3):e0013636. doi: 10.1371/journal.pntd.0013636 (PMC13052988; doi:10.1371/journal.pntd.0013636)
Supplement: S7 Table — (DOCX) [file pntd.0013636.s010.docx]

**S7 Table: Distribution of dengue Serotypes in Goa during 2019-2024**

| **Year** | **District** | **Den 1** | **Den 2** | **Den 3** | **Den 4** | **Mixed infection** |
| --- | --- | --- | --- | --- | --- | --- |
| 2019 | North Goa | 2 | 8 | 4 | 0 | 0 |
|  | South Goa | 0 | 6 | 5 | 3 | 0 |
| 2020 | North Goa | 14 | 13 | 29 | 0 | 0 |
|  | South Goa | 7 | 34 | 9 | 1 | 0 |
| 2021 | North Goa | 7 | 36 | 10 | 0 | 0 |
|  | South Goa | 10 | 18 | 1 | 1 | 0 |
| 2022 | North Goa | 3 | 58 | 0 | 0 | 0 |
|  | South Goa | 6 | 28 | 0 | 0 | 0 |
| 2023 | North Goa | 47 | 179 | 38 | 2 | 0 |
|  | South Goa | 28 | 8 | 13 | 2 | 0 |
| 2024 | North Goa | 22 | 12 | 7 | 4 | 0 |
|  | South Goa | 2 | 3 | 7 | 1 | 0 |
| **Total** | **North Goa** | **95** | **306** | **88** | **6** | **0** |
|  | **South Goa** | **53** | **97** | **35** | **8** | **0** |
