# Supplementary material for: Dengue transmission dynamics in an urban setting in western India
Source: PLoS Negl Trop Dis. 2026 Mar 23;20(3):e0013636. doi: 10.1371/journal.pntd.0013636 (PMC13052988; doi:10.1371/journal.pntd.0013636)
Supplement: S8 Table — (DOCX) [file pntd.0013636.s011.docx]

**S8 Table:** Seasonal Mann-Kendall test for monotonous trends in 34 health centres of Goa

| **District** | **Health Centre** | **Z Statistic** | **p-value** | **tau** |
| --- | --- | --- | --- | --- |
| North Goa | Aldona | 2.07 | 0.04 | 0.17 |
|  | Betki | -1.41 | 0.16 | -0.06 |
|  | Bicholim | 2.21 | 0.03 | 0.31 |
|  | Candolim | 2.80 | 0.01 | 0.22 |
|  | Cansarvanem | 2.05 | 0.04 | 0.20 |
|  | Chimbel | 1.90 | 0.06 | 0.21 |
|  | Colvale | 3.90 | <0.01 | 0.40 |
|  | Corlim | -2.10 | 0.04 | -0.13 |
|  | Mapusa | 4.00 | <0.01 | 0.40 |
|  | Mayem | 2.14 | 0.03 | 0.15 |
|  | Panaji | 1.07 | 0.28 | 0.10 |
|  | Pernem | 2.13 | 0.03 | 0.34 |
|  | Porvorim | 2.08 | 0.04 | 0.31 |
|  | Saligao | 1.81 | 0.07 | 0.27 |
|  | Sanquelim | 1.64 | 0.10 | 0.17 |
|  | Siolim | 3.80 | <0.01 | 0.41 |
|  | Valpoi | 0.40 | 0.69 | 0.02 |
| South Goa | Bali | -0.23 | 0.82 | -0.01 |
|  | Canacona | 1.04 | 0.30 | 0.07 |
|  | Cansaulim | -0.72 | 0.47 | -0.03 |
|  | Chinchinim | -1.68 | 0.09 | -0.06 |
|  | Cortalim | 2.06 | 0.04 | 0.17 |
|  | Curchorem | -0.95 | 0.34 | -0.04 |
|  | Curtorim | -0.41 | 0.69 | -0.03 |
|  | Dharbandora | 0.16 | 0.87 | 0.01 |
|  | Loutolim | -0.83 | 0.41 | -0.05 |
|  | Marcaim | -1.73 | 0.08 | -0.06 |
|  | Margao | 1.72 | 0.09 | 0.15 |
|  | Navelim | 1.61 | 0.11 | 0.09 |
|  | Ponda | -0.54 | 0.59 | -0.03 |
|  | Quepem | 1.66 | 0.10 | 0.08 |
|  | Sanguem | -0.19 | 0.85 | 0.00 |
|  | Shiroda | -1.28 | 0.20 | -0.10 |
|  | Vasco | 1.52 | 0.13 | 0.17 |
