# Supplementary material for: Dengue transmission dynamics in an urban setting in western India
Source: PLoS Negl Trop Dis. 2026 Mar 23;20(3):e0013636. doi: 10.1371/journal.pntd.0013636 (PMC13052988; doi:10.1371/journal.pntd.0013636)
Supplement: S9 Table — (DOCX) [file pntd.0013636.s012.docx]

**S9 Table:** Correlation Analysis of different meteorological variables with dengue cases in each of the 34 health centres in Goa

| ***PHC/UHC*** | ***Mean Temp.*** | ***Max Temp.*** | ***Min Temp.*** | ***Total Rainfall*** | ***Max Rainfall*** | ***Min Rainfall*** | ***Mean RH*** | ***Max RH*** | ***Min RH*** | ***Mean Atm. Press.*** | ***Max Atm. Press.*** | ***Min Atm. Press.*** | ***Mean Wind Speed*** | ***Max Wind Speed*** | ***Min Wind Speed*** |
| --- | --- | --- | --- | --- | --- | --- | --- | --- | --- | --- | --- | --- | --- | --- | --- |
| ***Aldona*** | *-0.10* | *-0.15* | *0.08* | *0.08* | *0.14* | *-0.04* | *0.17* | *0.13* | *0.11* | *0.00* | *-0.01* | *-0.04* | *-0.07* | *0.06* | *-0.15* |
|  | *0.24* | *0.08* | *0.33* | *0.37* | *0.10* | *0.67* | *0.04* | *0.11* | *0.18* | *0.96* | *0.91* | *0.62* | *0.41* | *0.44* | *0.07* |
| ***Bali*** | *0.04* | *0.10* | *0.00* | *-0.02* | *-0.01* | *-0.07* | *-0.11* | *-0.08* | *-0.03* | *0.02* | *0.02* | *-0.01* | *-0.05* | *-0.05* | *0.13* |
|  | *0.65* | *0.22* | *0.98* | *0.80* | *0.93* | *0.38* | *0.20* | *0.32* | *0.73* | *0.78* | *0.85* | *0.92* | *0.52* | *0.54* | *0.13* |
| ***Betki*** | *-0.12* | *-0.24* | *0.14* | *0.23* | *0.21* | *0.22* | *0.20* | *0.23* | *0.15* | *-0.16* | *-0.16* | *-0.10* | *0.22* | *0.16* | *-0.06* |
|  | *0.14* | *0.00* | *0.10* | *0.01* | *0.01* | *0.01* | *0.01* | *0.00* | *0.06* | *0.05* | *0.05* | *0.21* | *0.01* | *0.06* | *0.48* |
| ***Bicholim*** | *-0.01* | *-0.13* | *0.15* | *0.05* | *0.15* | *-0.04* | *0.17* | *0.15* | *0.15* | *0.01* | *-0.02* | *-0.05* | *-0.02* | *0.02* | *-0.04* |
|  | *0.90* | *0.11* | *0.07* | *0.53* | *0.08* | *0.62* | *0.04* | *0.08* | *0.07* | *0.89* | *0.79* | *0.58* | *0.77* | *0.82* | *0.60* |
| ***Canacona*** | *0.01* | *0.01* | *0.09* | *0.07* | *0.10* | *-0.02* | *0.03* | *0.00* | *0.01* | *0.05* | *0.04* | *0.03* | *0.00* | *0.29* | *0.09* |
|  | *0.88* | *0.90* | *0.29* | *0.39* | *0.25* | *0.82* | *0.77* | *0.98* | *0.91* | *0.53* | *0.60* | *0.77* | *0.98* | *0.00* | *0.26* |
| ***Candolim*** | *-0.03* | *-0.09* | *0.11* | *0.17* | *0.22* | *0.01* | *0.16* | *0.14* | *0.17* | *-0.08* | *-0.10* | *-0.14* | *0.06* | *0.42* | *-0.03* |
|  | *0.71* | *0.29* | *0.21* | *0.04* | *0.01* | *0.93* | *0.05* | *0.09* | *0.04* | *0.36* | *0.24* | *0.09* | *0.45* | *0.00* | *0.74* |
| ***Cansarvanem*** | *-0.13* | *-0.20* | *0.08* | *0.17* | *0.20* | *0.03* | *0.22* | *0.19* | *0.16* | *-0.04* | *-0.05* | *-0.05* | *0.03* | *0.00* | *-0.24* |
|  | *0.14* | *0.02* | *0.31* | *0.04* | *0.02* | *0.71* | *0.01* | *0.02* | *0.05* | *0.62* | *0.55* | *0.53* | *0.75* | *0.97* | *0.00* |
| ***Cansaulim*** | *-0.02* | *0.02* | *-0.05* | *0.00* | *-0.01* | *0.00* | *-0.01* | *0.00* | *0.02* | *0.05* | *0.05* | *0.00* | *-0.04* | *0.11* | *0.08* |
|  | *0.84* | *0.78* | *0.59* | *0.98* | *0.86* | *1.00* | *0.91* | *0.97* | *0.81* | *0.56* | *0.58* | *0.97* | *0.65* | *0.20* | *0.36* |
| ***Chimbel*** | *0.00* | *-0.13* | *0.19* | *0.19* | *0.26* | *0.01* | *0.24* | *0.18* | *0.19* | *-0.09* | *-0.11* | *-0.11* | *0.07* | *0.08* | *-0.30* |
|  | *0.96* | *0.13* | *0.02* | *0.03* | *0.00* | *0.92* | *0.00* | *0.03* | *0.02* | *0.29* | *0.18* | *0.20* | *0.43* | *0.37* | *0.00* |
| ***Chinchinim*** | *0.03* | *0.10* | *-0.04* | *-0.05* | *-0.03* | *-0.05* | *-0.08* | *-0.08* | *-0.10* | *0.11* | *0.07* | *0.06* | *-0.08* | *0.16* | *0.17* |
|  | *0.69* | *0.22* | *0.62* | *0.52* | *0.70* | *0.56* | *0.35* | *0.31* | *0.24* | *0.18* | *0.41* | *0.46* | *0.34* | *0.05* | *0.04* |
| ***Colvale*** | *-0.02* | *-0.11* | *0.09* | *0.11* | *0.11* | *0.02* | *0.15* | *0.11* | *0.06* | *-0.03* | *-0.05* | *-0.08* | *0.01* | *-0.04* | *-0.19* |
|  | *0.81* | *0.19* | *0.26* | *0.17* | *0.18* | *0.83* | *0.08* | *0.17* | *0.47* | *0.68* | *0.57* | *0.33* | *0.87* | *0.67* | *0.02* |
| ***Corlim*** | *0.09* | *-0.07* | *0.18* | *0.02* | *0.06* | *0.04* | *0.15* | *0.12* | *0.15* | *-0.15* | *-0.13* | *-0.09* | *-0.01* | *0.14* | *0.00* |
|  | *0.26* | *0.41* | *0.03* | *0.78* | *0.50* | *0.65* | *0.08* | *0.15* | *0.07* | *0.07* | *0.13* | *0.30* | *0.93* | *0.09* | *0.96* |
| ***Cortalim*** | *-0.01* | *-0.13* | *0.13* | *0.19* | *0.19* | *0.09* | *0.18* | *0.14* | *0.19* | *-0.08* | *-0.07* | *-0.09* | *0.14* | *0.14* | *-0.23* |
|  | *0.89* | *0.12* | *0.11* | *0.02* | *0.03* | *0.29* | *0.03* | *0.09* | *0.02* | *0.35* | *0.37* | *0.30* | *0.09* | *0.10* | *0.01* |
| ***Curchorem*** | *-0.14* | *-0.17* | *0.08* | *0.17* | *0.11* | *0.18* | *0.13* | *0.10* | *0.10* | *-0.06* | *-0.08* | *-0.02* | *0.11* | *0.11* | *-0.02* |
|  | *0.10* | *0.04* | *0.32* | *0.04* | *0.21* | *0.03* | *0.13* | *0.22* | *0.21* | *0.50* | *0.33* | *0.80* | *0.20* | *0.20* | *0.80* |
| ***Curtorim*** | *0.01* | *-0.13* | *0.13* | *0.11* | *0.05* | *0.13* | *0.14* | *0.15* | *0.16* | *-0.07* | *-0.09* | *-0.07* | *0.11* | *0.19* | *-0.05* |
|  | *0.94* | *0.13* | *0.12* | *0.20* | *0.56* | *0.12* | *0.09* | *0.07* | *0.05* | *0.39* | *0.31* | *0.40* | *0.19* | *0.03* | *0.58* |
| ***Dharbandora*** | *0.04* | *-0.12* | *0.16* | *0.11* | *0.16* | *0.15* | *0.13* | *0.14* | *0.09* | *-0.09* | *-0.11* | *-0.06* | *0.16* | *0.15* | *-0.03* |
|  | *0.67* | *0.14* | *0.06* | *0.20* | *0.05* | *0.08* | *0.13* | *0.09* | *0.27* | *0.30* | *0.17* | *0.51* | *0.05* | *0.07* | *0.76* |
| ***Loutolim*** | *-0.12* | *-0.19* | *0.07* | *0.13* | *0.09* | *0.17* | *0.18* | *0.20* | *0.11* | *-0.08* | *-0.09* | *-0.05* | *0.08* | *0.14* | *-0.02* |
|  | *0.15* | *0.02* | *0.43* | *0.11* | *0.30* | *0.04* | *0.03* | *0.02* | *0.21* | *0.32* | *0.27* | *0.55* | *0.32* | *0.09* | *0.77* |
| ***Mapusa*** | *-0.01* | *-0.05* | *0.14* | *0.09* | *0.17* | *-0.06* | *0.18* | *0.10* | *0.10* | *0.00* | *-0.02* | *-0.07* | *-0.10* | *0.06* | *-0.19* |
|  | *0.91* | *0.55* | *0.10* | *0.30* | *0.04* | *0.50* | *0.03* | *0.25* | *0.22* | *0.98* | *0.83* | *0.38* | *0.24* | *0.45* | *0.02* |
| ***Marcaim*** | *-0.04* | *-0.07* | *0.07* | *0.19* | *0.33* | *0.15* | *0.09* | *0.08* | *0.06* | *-0.14* | *-0.13* | *-0.09* | *0.13* | *0.14* | *0.00* |
|  | *0.63* | *0.39* | *0.42* | *0.03* | *0.00* | *0.08* | *0.26* | *0.32* | *0.50* | *0.09* | *0.13* | *0.30* | *0.11* | *0.10* | *0.99* |
| ***Margao*** | *0.01* | *-0.15* | *0.20* | *0.15* | *0.17* | *0.10* | *0.20* | *0.19* | *0.18* | *-0.07* | *-0.10* | *-0.08* | *0.08* | *0.16* | *-0.09* |
|  | *0.86* | *0.06* | *0.02* | *0.07* | *0.04* | *0.21* | *0.01* | *0.03* | *0.03* | *0.44* | *0.24* | *0.31* | *0.35* | *0.05* | *0.27* |
| ***Mayem*** | *-0.07* | *-0.22* | *0.15* | *0.16* | *0.16* | *0.04* | *0.22* | *0.22* | *0.15* | *-0.08* | *-0.12* | *-0.05* | *0.08* | *0.08* | *-0.20* |
|  | *0.41* | *0.01* | *0.07* | *0.06* | *0.05* | *0.63* | *0.01* | *0.01* | *0.07* | *0.35* | *0.16* | *0.53* | *0.34* | *0.36* | *0.02* |
| ***Navelim*** | *0.06* | *0.04* | *0.04* | *0.02* | *0.07* | *-0.05* | *0.00* | *-0.02* | *-0.03* | *0.07* | *0.04* | *0.01* | *-0.10* | *0.07* | *0.09* |
|  | *0.49* | *0.63* | *0.60* | *0.80* | *0.38* | *0.54* | *0.96* | *0.77* | *0.74* | *0.37* | *0.67* | *0.88* | *0.22* | *0.40* | *0.28* |
| ***Panaji*** | *-0.09* | *-0.18* | *0.11* | *0.10* | *0.15* | *0.01* | *0.24* | *0.21* | *0.13* | *-0.11* | *-0.10* | *-0.17* | *0.03* | *0.06* | *-0.20* |
|  | *0.29* | *0.03* | *0.18* | *0.23* | *0.07* | *0.88* | *0.00* | *0.01* | *0.12* | *0.17* | *0.22* | *0.05* | *0.70* | *0.49* | *0.01* |
| ***Pernem*** | *0.04* | *-0.06* | *0.15* | *0.10* | *0.12* | *-0.04* | *0.13* | *0.09* | *0.07* | *-0.03* | *-0.01* | *-0.09* | *-0.05* | *0.09* | *-0.16* |
|  | *0.66* | *0.47* | *0.08* | *0.26* | *0.15* | *0.66* | *0.11* | *0.30* | *0.38* | *0.70* | *0.91* | *0.29* | *0.58* | *0.30* | *0.05* |
| ***Ponda*** | *0.03* | *-0.15* | *0.14* | *0.12* | *0.04* | *0.16* | *0.14* | *0.15* | *0.10* | *-0.11* | *-0.15* | *-0.07* | *0.13* | *0.08* | *-0.05* |
|  | *0.70* | *0.08* | *0.09* | *0.15* | *0.68* | *0.05* | *0.11* | *0.07* | *0.23* | *0.17* | *0.08* | *0.39* | *0.13* | *0.35* | *0.55* |
| ***Porvorim*** | *-0.10* | *-0.07* | *0.00* | *0.01* | *-0.01* | *-0.02* | *0.04* | *0.01* | *0.02* | *0.12* | *0.08* | *0.08* | *-0.03* | *-0.01* | *-0.06* |
|  | *0.25* | *0.41* | *0.99* | *0.94* | *0.92* | *0.81* | *0.61* | *0.87* | *0.78* | *0.14* | *0.31* | *0.31* | *0.68* | *0.94* | *0.50* |
| ***Quepem*** | *0.00* | *0.04* | *-0.05* | *-0.02* | *0.05* | *-0.02* | *-0.01* | *-0.04* | *-0.02* | *0.12* | *0.06* | *0.09* | *-0.08* | *-0.03* | *0.15* |
|  | *0.97* | *0.60* | *0.56* | *0.84* | *0.53* | *0.82* | *0.91* | *0.62* | *0.82* | *0.17* | *0.50* | *0.29* | *0.32* | *0.74* | *0.08* |
| ***Saligao*** | *-0.02* | *-0.07* | *0.10* | *0.04* | *0.05* | *-0.03* | *0.14* | *0.10* | *0.06* | *0.01* | *-0.04* | *0.03* | *0.00* | *-0.05* | *-0.28* |
|  | *0.78* | *0.40* | *0.23* | *0.63* | *0.51* | *0.72* | *0.09* | *0.21* | *0.46* | *0.86* | *0.66* | *0.72* | *0.98* | *0.55* | *0.00* |
| ***Sanguem*** | *0.03* | *-0.11* | *0.14* | *0.12* | *0.08* | *0.18* | *0.12* | *0.12* | *0.13* | *-0.02* | *-0.03* | *-0.06* | *0.12* | *0.11* | *0.00* |
|  | *0.74* | *0.18* | *0.10* | *0.14* | *0.34* | *0.03* | *0.14* | *0.16* | *0.14* | *0.79* | *0.72* | *0.48* | *0.15* | *0.19* | *0.97* |
| ***Sanquelim*** | *-0.03* | *-0.05* | *0.09* | *0.02* | *0.03* | *-0.06* | *0.08* | *0.05* | *0.05* | *0.07* | *-0.01* | *0.11* | *-0.02* | *-0.01* | *-0.06* |
|  | *0.76* | *0.54* | *0.30* | *0.78* | *0.68* | *0.51* | *0.34* | *0.53* | *0.53* | *0.40* | *0.94* | *0.21* | *0.80* | *0.86* | *0.48* |
| ***Shiroda*** | *-0.08* | *-0.07* | *0.02* | *0.03* | *0.01* | *0.00* | *0.00* | *0.01* | *-0.02* | *0.11* | *0.06* | *0.04* | *-0.03* | *0.18* | *0.15* |
|  | *0.34* | *0.43* | *0.86* | *0.72* | *0.86* | *0.98* | *0.97* | *0.89* | *0.79* | *0.18* | *0.45* | *0.62* | *0.72* | *0.04* | *0.07* |
| ***Siolim*** | *-0.05* | *-0.08* | *0.12* | *0.12* | *0.12* | *-0.01* | *0.21* | *0.12* | *0.10* | *-0.03* | *-0.05* | *-0.08* | *-0.07* | *-0.01* | *-0.30* |
|  | *0.59* | *0.35* | *0.16* | *0.15* | *0.14* | *0.90* | *0.01* | *0.16* | *0.24* | *0.68* | *0.57* | *0.32* | *0.40* | *0.92* | *0.00* |
| ***Valpoi*** | *0.00* | *-0.11* | *0.13* | *0.14* | *0.19* | *0.14* | *0.11* | *0.12* | *0.10* | *-0.17* | *-0.21* | *-0.10* | *0.18* | *0.16* | *-0.09* |
|  | *1.00* | *0.20* | *0.13* | *0.10* | *0.02* | *0.11* | *0.18* | *0.16* | *0.23* | *0.04* | *0.01* | *0.24* | *0.03* | *0.05* | *0.30* |
| ***Vasco*** | *-0.08* | *-0.14* | *0.07* | *0.13* | *0.14* | *0.04* | *0.20* | *0.14* | *0.20* | *-0.08* | *-0.06* | *-0.07* | *0.03* | *0.05* | *-0.23* |
|  | *0.34* | *0.09* | *0.40* | *0.13* | *0.10* | *0.66* | *0.02* | *0.09* | *0.02* | *0.32* | *0.47* | *0.41* | *0.68* | *0.56* | *0.01* |
