# Supplementary material for: Dengue transmission dynamics in an urban setting in western India
Source: PLoS Negl Trop Dis. 2026 Mar 23;20(3):e0013636. doi: 10.1371/journal.pntd.0013636 (PMC13052988; doi:10.1371/journal.pntd.0013636)
Supplement: S10 Table — (DOCX) [file pntd.0013636.s013.docx]

**S10 Table:** Deviation Information Criterion (DIC) and the performance log score of the single variable models.

| **Model** | **DIC** | **log score** |
| --- | --- | --- |
| Avg Temperature Model | 13175 | 1.69 |
| Min Temperature Model | 13178 | 1.694 |
| Max Temperature Model | 13176 | 1.691 |
| Max Atm Pressure Model | 13173 | 1.681 |
| Min Relative Humidity Model | 13134 | 1.599 |
| Total Precipitation Model | 11024 | 1.317 |
| Avg Wind Speed Model | 13176 | 1.69 |
