# Supplementary material for: Dengue transmission dynamics in an urban setting in western India
Source: PLoS Negl Trop Dis. 2026 Mar 23;20(3):e0013636. doi: 10.1371/journal.pntd.0013636 (PMC13052988; doi:10.1371/journal.pntd.0013636)
Supplement: Attachment — Submitted filename: Comments.docx [file pntd.0013636.s014.docx]

**REPLY TO REVIEWER’S COMMENTS**

**PNTD-D-25-01818**

Dengue transmission dynamics in an urban setting in western India by K Balasubramani et al.

Dear reviewers,

On behalf of all the authors, I extend my sincere gratitude for your valuable time and effort spent to improve the quality of the manuscript. We have thoroughly reviewed each recommendation and addressed all queries in the revised manuscript. We appreciate the reviewers’ attention to detail in their corrections and suggestions, particularly in the methodology section. The revisions have been clearly highlighted (track changes) in the attached manuscript to facilitate your final review. We believe these changes have resolved previous concerns and are now much more robust, providing a clearer contribution to the field.

**Point-by-point response to Reviewers’ comments**

Dengue is emerging in many parts of India, and there is a need to assess its risk using modern tools, including time-series analysis, for early forecasting and timely response. This study is therefore very relevant and addresses these needs in urban centres, both in the coastal zones in western India and elsewhere in the countries with similar ecologies.

This manuscript needs improvement for better readability. I have listed many suggestions below.

**Abstract**

On line 31: Replace “is critical’ with “was undertaken”

**Response:** The phrase *“is critical”* has been replaced with *“was undertaken”* (line no. 31).

Line 35: Change “surveillance data” to “passive surveillance data routinely collected from 34 health facilities.”

**Response:** “*surveillance data*” has been revised to “*passive surveillance data routinely collected from 34 health facilities*” (line no. 35).

Line 37: Change “clusters of dengue” to “clusters of dengue cases”

**Response:** *“clusters of dengue”* has been corrected to *“dengue case clusters”* for better flow of sentence (line no. 38).

Line 43: Change “serotype” to “dengue virus serotype”—mention which other serotypes were also reported.

**Response:** “*serotype*” has been changed to “*dengue virus serotype*”, and other serotypes are now mentioned.

*“The dominant dengue virus serotype in Goa was DEN-2, accounting for 58.57% of infections, followed by DENV 1 (21.51%), DENV 3 (17.87.%), and DENV 4 (2.03%)”* (line no. 44 – 45)

Line 44: Modify this sentence to “The clusters of dengue cases were predominantly observed in North Goa— the SMK test showed a significant positive trend for dengue cases in almost all the [give number] health facilities in the North Goa district.

**Response:** The modification in the sentence has now been made

*“The clusters of dengue cases were predominantly observed in North Goa— the SMK test showed a significant positive trend for dengue cases across 16 of the 17 health facilities in the North Goa district.”*(line no. 45-47)

Line 46: Change “show” to “showed”

**Response:** The word *“show”* has been changed to *“showed”*(line no. 46).

Line 47: Change to “clusters in the PHCs Candolim, ...”

**Response:** The text has been revised to *“clusters in the health centres*…” in line 49.

Line 51: Change “rainfall threshold of 630-607” to “total annual rainfall of 607 to 630 mm.”

**Response:** The phrase *“rainfall threshold of 630–607”* has been corrected to *“total annual rainfall of 607mm...”*(line no. 53).

Line 57: “early warning for dengue in Goa”

**Response:** The phrase *“early warning for dengue in Goa”* is incorporated in the revised manuscript (line no. 59).

**Introduction**

Lines 87-88: Update the names of the states reporting the most dengue cases and deaths in India according to the data available from the NCVBDC website from <https://ncvbdc.mohfw.gov.in/index4.php?lang=1&level=0&linkid=431&lid=3715>

**Response**: We have now updated the states as per the dengue case load in 2024

*“…with most cases reported from Karnataka, Tamil Nadu, Kerala, Maharashtra, Uttar Pradesh, Rajasthan, Delhi, Madhya Pradesh, Bihar and Telangana in 2024 [8,14].”* (line no. 88-89).

Lines 98-99: Instead of reporting cases in 2019 in Goa, update the dengue case numbers in the last 2-3 years in Goa (e.g. to approximately 600 cases and 3 deaths in 2024, or quote updated statistics from the Goa state sources).

**Response:** As suggested by the reviewer, the correction has been made and updated with 2024 data.

*“… the past two decades, its burden has increased steadily in the state, with 567 cases and 3 deaths reported in 2024 [14].”* (line no. 99-100).

Line 101: Cite more recent estimates of the economic burden of dengue (e.g. 2016 by Shepard et al. (Lancet Infect Dis. 2016 Aug;16(8):935-41. doi: 10.1016/S1473-3099(16)00146-8).

**Response:** Cited Shepard DS, Halasa YA, Tyagi BK, Adhish SV, Nandan D, Karthiga KS, et al. Economic and Disease Burden of Dengue Illness in India. Am J Trop Med Hyg. 2014;91: 1235–1242. doi:10.4269/ajtmh.14-0002 (line no. 102).

**Methodology**

Line 125-129: How many health facilities (PHCs, UHCs, CHCs) are there in total, and did the collected data on dengue cases cover all of them?

**Response:** A total of 34 government health facilities (PHCs, UHCs, CHCs) are there in total, and the collected data on dengue cases covered all of them.

***“****The annual dengue case records from 2011 to 2024 were sourced from all the 34 health centers across Goa: 24 Primary Health Centers (PHCs), 04 Urban Health Centers (UHCs), and 06 Community Health Centers (CHCs)…”.* (line no. 126-128).

Line 127-128: Replace “Age sex wise data” with “data by age and sex”. Is NVBDCP still the correct acronym?

**Response:** The phrase *“Age sex wise data”* is now replaced with *“data by age and sex”* age and sex wise data*.* The acronym NVBDCP has been retained, as the data was sourced from the NVBDCP, Directorate of Health Services (DHS), Goa (line no. 129).

Lines 130-131: Clarify for which years the data were obtained/analysed (note: Reference # 16 was published in 2020).

**Response:** The years for the meteorological datasets have been added to the manuscript. The reference describes the original publication of these datasets, and since then they are regularly updated with newer data available at the European Centre for Medium Range Weather Forecasts’ Copernicus Climate Data store (line no. 137).

Lines 133-134: The meteorological data source is from the year 2020 and the study period was 2011–2024, so how was it possible that the meteorological variables were computed for the period beyond 2020 when no data were obtained?

**Response:** The reference describes the original publication of these datasets, and since then they are regularly updated with newer data available at the European Centre for Medium Range Weather Forecasts’ Copernicus Climate Data store.

Line 139: Put “S1 and S2 Tables” in brackets.

**Response:** “S1 and S2 Tables” have been placed within brackets (line no. 147).

Line 140: Use lowercase text in the subtitle.

**Response:** The subtitle text has been changed to lowercase (line no. 154).

Line 151: Use lowercase text in the subtitle.

**Response**: The subtitle text has been changed to lowercase (line no. 170).

Line 152: It is better to state “The dengue data by health facilities or health centres” because data were collected from the UHCs and CHCs as well. Later, you also mentioned PHCs only (e.g., lines 154, 157, 159, and so on).

**Response:** The phrase has been revised to *“health centers”* to accurately reflect data collection from PHCs, UHCs, and CHCs, and terminology has been made consistent throughout the manuscript (line no. 171).

Line 153: “location (Lat, Long)” to “geographical coordinates”.

**Response:** *“location (Lat, Long)”* has been replaced with *“geographical coordinates”* (line no. 172)*.*

Line 198: Use lowercase text in the subtitle.

**Response:** The subtitle text has been changed to lowercase (line no. 245).

Line 217: Use lowercase text in the subtitle.

**Response:** The subtitle text has been changed to lowercase (line no. 285).

Line 234: Use lowercase text in the subtitle. Here, you have rightly used the term “health centres”, so maintain uniformity.

**Response:** The subtitle text has been changed to lowercase, and the term *“health centres”* has been retained to ensure uniform terminology (line no. 304).

**Results**

There is no mention of the number of dengue deaths in Goa. The national programme reported just 7 deaths in 2022-2024 (<https://ncvbdc.mohfw.gov.in/index4.php?lang=1&level=0&linkid=431&lid=3715>). Check the local health facility data to see if reporting of any deaths was missed.

**Response:** The number of dengue deaths in Goa, between 2011-2024, has now been mentioned as well as added to (S5 Table).

“*between 2011-2024, 12 deaths due to dengue were recorded……..”*(line no. 305).

Lines 238-240: In this sentence ending with “reported in North Goa”, insert “(**S5 Table**)” to inform the reader availability of detailed data in S5 Table. Delete “S5 Table” in line 248.

**Response:** Corrected as per the suggestion (line no. 305).

Line 240: By “>100” do you mean “>100 cases per health facility”?

**Response:** The sentence is now reframed for clarity

*“…reported >100 dengue cases.”*(line no. 313)

Lines 240-244: This long sentence needs clarity– are you talking of centres located in two districts?

**Response:** The sentence mentions the list of health centres reporting >100 dengue cases in North and South Goa districts. The sentence is now reframed for clarity

*“Line 245-249: Candolim PHC, Mapusa UHC, Colvale PHC, Siolim PHC, Aldona PHC, Valpoi CHC, Sanquelim CHC, Porvorim PHC, Pernem CHC, Saligao PHC, and Panaji UHC in North Goa District, and Vasco UHC, Margao UHC, Shiroda PHC, Curtorim PHC, Canacona CHC, and Cortalim PHC, in the South Goa District reported >100 dengue cases”* (line no. 309-313).

**Fig. 1A:** Split this single Figure 1A into three different figures since they convey different messages.

**Response:** Figure 1 is separated into two figures (Figure 1 and Figure 2). Figure 1B and 1C are not separated as already the manuscript contains more (eight) figures (line no. 318).

Line 255: Modify to “Mean monthly distribution ....”.

**Response:** Thank you for the suggestion, but we have used the total monthly dengue cases from 2012 to 2024.

Lines 259- 260: The total number of cases was 4717 (see line 245 and S5 Table), so 61.49% cannot be just 1335 cases. Something is amiss here.

**Response:** We agree with the reviewer that the total number of cases was 4717. However, it may be noted that the 4717 cases represent the total 2011-2024 study period.

*“alone accounted for 82.14% (3,875) of the total dengue cases (4,717) between 2011 and 2024.”* (line no. )

The (61.49%; 1335 cases) only represents the age and sex wise distribution of dengue cases for the last four years (2021-2024) dataset. This is also addressed in the results. *‘‘**The age and sex wise distribution of dengue cases in the last four years (2021-2024) shows the majority (61.49%; 1335 cases)’’* (line no. 327)*.*

Line 260: Also, most cases in ≥ 15-year-olds could probably be because they constituted a majority in the total population. Without denominators (the total number of people in each age and sex group within the population being studied), you cannot calculate accurate rates or proportions.

**Response:** Age- and sex-wise population data were not available. Hence, we did not calculate rates or proportions. However, we showed the distribution of cases with different age group (below 15 years and 15 years and above) and sex (line no. 327).

Lines 262-264: In **Figure 1B**, what do the two different colours of data bars (blue and red) indicate?

**Response:** The bars in the graph represent the cumulative number of dengue cases reported in 2012-2024. The colours blue and red were used to highlight the increase in dengue cases from June to December. The sentence is now updated for clarity.

*“Blue and red colour gradients represent lower caseloads during January–May and high caseloads during the rainy (monsoon) season of June–December, respectively”* (line no. 338)*.*

Line 269-270: During 2020-2023, DENV-2 was the predominant serotype. In 2024, when only about 50 serotypes were reported, DENV-1 may have predominated. So, this does not indicate a rising trend for DENV-1.

**Response:** We agree with the reviewer’s comment, and we have now toned down the sentence. *“Line 275-276: Particularly in 2024, DENV 1 serotype accounted for 41.37% of the samples (Fig 2B)”* (line no. 336)*.*

Line 274-275: In this sentence, you mention **cumulative incidence per 100,000 persons**, but Table S5 presents dengue cases and not incidence. This is confusing and needs clarity.

**Response:** We thank the reviewer for pointing out this inconsistency. Table S5 has been revised to improve clarity and now includes the estimated population, annual dengue incidence per 100,000 person-years for Goa state, and the average annual incidence per 100,000 person-years for each health center in Goa. The text in Lines 274–275 has been updated accordingly to ensure consistency between the manuscript and the supplementary table (line no. 344).

Line 286: Use lowercase text in the subtitle.

**Response:** The subtitle text has been changed to lowercase (line no. 359).

Line 342: After the sentence “the most southern health centers would remain with < 20 cases”, clarify that the southernmost Canacona health centre might report a caseload of >40.

Response: As suggested, the sentence has now been modified

*“Line 348-349: southern centers remain < 20 cases except Canacona CHC that reported a caseload of >40”* (line no. 416)*.*

Line 372: Use lowercase text in the subtitle.

**Response:** The subtitle text has been changed to lowercase (line no. 449).

Line 377: It will be more logical to state that “The high incidence of dengue followed high rainfall.” Also, is it “temperature” or “mean monthly temperature”?

Fig. 6 and lines 394 and later: Replace “Precipitation” with “Rainfall” since the term precipitation may include snowfall, which does not happen in Goa.

**Response:** As suggested by the reviewer, the sentence has now been modified

“Line 384-385: in Goa. The high incidence of dengue is followed by high rainfall. In contrast, the monthly average temperature remains ambient throughout the year in both North and South Goa”, and throughout the manuscript (including line 394 and subsequent references), the term ***“Precipitation”*** has been replaced with *“Rainfall”*, as snowfall does not occur in the study region (line no. 453-455).

Line 385: Do you mean to say that in some of the health centres, there was a positive correlation between rainfall and dengue cases? The pooling of health centre data to get a district trend (in Fig. 6) might have possibly lost a positive association. You may possibly check the correlation between the amount of rainfall in June-July (early rains) and the cases.

**Response:** Correlation test between dengue cases and climatic variables, particularly rainfall did not yield any significant positive correlations at the district or PHC level. This is likely because, as shown in Figure 6, the relation between rainfall and dengue incidence is likely a lagged effect, which cannot be captured in correlation tests only. The correlation test was simply done to obtain an initial understanding about the relationships. Association between climatic variables with dengue cases was more clearly assessed using the DLNM model, which also incorporated the lagged effects, and the results of these models do indeed show a very strong positive association of rainfall with dengue incidence (line no. 462).

Line 401: Replace “total precipitation” with “The total annual rainfall...”

**Response:** Replaced *“total precipitation”* with *“The total annual rainfall...”* (line no. 484)*.*

Line 407: What is LU/LC – land use/land use class? Try to minimise the use of abbreviations/acronyms to improve the readability of the text unless a term appears frequently such as more than 6 times in the manuscript).

Response: LU/LC is a standard abbreviation for land use/land cover mentioned in *line no. 139* (line no. 509).

**Discussion**

Line 425: Which year is correct – 2001 (see line 97) or 2011?

**Response:** 2001 is the correct year and has been corrected accordingly (line no. 518).

Line 430: More correctly, the upward trend was after 2020 and not 2012.

**Response:** The correction is made in the manuscript (line no. 522).

Line 433-434: Revisit this sentence in view of an earlier comment. If this inference is found true, discuss possible reasons, such as higher occupational exposures of adult males or something else .

**Response:** The male predominance may be attributed to gender based factors like high occupational exposure and differences in clothing, studies have also reported the incidence of dengue more in males than in females (<https://www.tandfonline.com/doi/full/10.1080/23744235.2025.2528132#d1e340>) (line no. 55).

Line 436-438: “Goa is also seeing the emergence of DENV-3 as a dominant serotype” is incorrect. The dominant serotype has remained DENV-2, although you may state that all four serotypes were cocirculating in all the years of data analysis (2019–2024; see Fig. 1B).

**Response:** The sentence is now been revised as follows

*“**Line 442-443: In addition to DENV-1 and DENV-2, Goa is also witnessing an increase in DENV-3 serotype.”* (line no. 527-528)

Line 444-445: Absence of mixed serotypes also indicates a low grade of transmission of DENV-1, 3 and 4.

Discuss the reporting of just a few dengue deaths in the last 3-4 years.

**Response:** Acknowledged the limitation as per the suggestion

Line 642-644: *“**The serotypes were tested on a limited sample, which may not accurately reflect the actual distribution of serotypes in the state, and the presence of mixed serotype infections couldn’t be captured.”* (line no. 652-654)

Line 508-516: In addition to serosurveys in the general population, would you also suggest conducting a study to assess the role of migrant labourers in carrying the dengue virus with different serotypes?

**Response:** Thank you for the suggestion. We have added it in the revised conclusion

Line 666-667: *“Strengthening both passive surveillance and active case detection of cases, which will be crucial for improving the accuracy of disease burden, which can aid in controlling the disease transmission at health centre level.* *Screening for dengue cases from migrant workers will also reduce importation of dengue cases from other endemic states.”* (line no. 667-671)

Line 518-519: Correct to total annual rainfall.

**Response:** Corrected to total annual rainfall.

Line 529: Can you throw some light on how cases were diagnosed at the health centres – by NS1/RDT or other, and if non-availability of diagnostic facilities contributed to underreporting?

**Response:** the cases at the health centres were diagnosed by MAC-ELISA.

**References**

Format references according to the journal’s style of references.

Italicise species names.

**Response:** species names in the references are now Italicised

Reference # 1: Change KUMARA to lowercase.

**Response:** Corrected

Reference # 13: correct the typo “Dengue casea” to “Dengue cases”

**Response:** Corrected

Reference # 14: correct the typo “019 to “2019”

**Response:** Corrected

## **Title: Dengue transmission dynamics in an urban setting in western India**

This study presents an important contribution to understanding dengue epidemiology with comprehensive data analysis that holds relevance for public health efforts in Goa. However, to further enhance the study’s impact, it would be helpful to strength some presentation aspects and provide a more integrated interpretation of the results. This could include adjustments for population size, clearer explanations of statistical methods, consideration of spatial and temporal dependencies, and transparent reporting of uncertainties. Taking these steps would strengthen the scientific rigor and improve the practical value of the finding of dengue control.

1. The study depends on raw dengue case counts without adjusting for population size or catchment variation. Could this affect the validity of comparing disease risk across different times and locations? It would be beneficial to adjust dengue case counts for population size or catchment area differences. This adjustment allows for a more accurate and meaningful comparison of dengue risk over time and space, ensuring that variations in case counts reflect true differences in disease burden rather than simply differences in population size.

**Response:** We agree with the reviewer. The normalization of cases with the total population allows for a more meaningful comparison of dengue risk over time and space. However, the updated population numbers for the catchment areas of the health centres are not accurately available. As the last Census was conducted in 2011, all the population statistics for Goa (and India) are estimated based on the Census 2011. Moreover, the health centres are designed/located in such a way that each centre has a catchment population of ~30000 to 50000. Therefore, we believe the total cases in each of these health centres will give important insights into the overall trend of dengue transmission. However, as requested, we have also calculated the incidence of dengue cases based on the estimated population data, and we have given the average annual incidence for all health centres and annual incidence for the state of Goa in supplementary file 5 (S5 Table. Dengue incidence data from 2011 to 2024 in the state of Goa.)

2. The analysis uses only passive surveillance data, which often misses many dengue cases in India. Have the authors thought about adjusting their models to correct for underreporting? Using methods like expansion factors from active surveillance, capture-recapture, or model-based corrections can give more accurate estimates and improve cluster detection and forecasting. Without such adjustments, the results probably underestimate the real dengue burden and may be biased.

**Response:** We agree with the reviewer’s comment. In this study, the analysis relied solely on passive surveillance data from health centres (PHCs, UHCs, and CHCs), which may underestimate the real dengue burden and be biased. Data required to apply expansion factors from active surveillance, capture-recapture, or model-based corrections were not available for Goa. Therefore, we were unable to adjust for underreporting, which has been acknowledged as a limitation of the study. Our findings should therefore be interpreted as representing the reported dengue case trend rather than the absolute incidence of dengue in Goa. Since the same surveillance system was consistently used across all locations throughout the study period, the analysis remains useful for understanding relative spatiotemporal patterns at the health centre level, identifying clusters with high case reports, and informing programmatic decision-making.

*“The data rely only on passive surveillance, which is* *prone to substantial under-reporting, and many individuals with mild or asymptomatic conditions do not seek medical attention. The woeful underreporting of dengue cases in India has also been highlighted in earlier studies [10]. This could lead to underestimation of absolute dengue burden in the state and may affect the precision of risk estimates and forecasting outputs, particularly in areas with variable healthcare access or diagnostic practices.”* (line 636-641)

We have clarified this in the revised manuscript, noting that incorporating active surveillance data into future work would allow for more accurate burden estimation.

*“**Future research should focus on integrating spatiotemporal climatic models with real-time case data and vector indices, using machine learning to build operational early warning tools.”* (line 676-678)

3. The paper uses secondary data for dengue serotypes but doesn’t give enough details about how samples were collected or tested. The analysis excludes mixed infections, which can affect disease spread and severity. Can the authors explain why they left these out? If the serotypes in mixed infections are known, they should be included to give a fuller picture.

**Response:** We appreciate the reviewer's point. The dengue serotype data used in the study were derived from secondary laboratory records. A total of 495 samples from North Goa and 193 from South Goa, collected between 2019 and 2024, were screened for dengue serotypes using RT PCR. In the available dataset, no mixed serotype infections were detected; therefore, none were included in the analysis. This is mentioned in the results section.

***“****From 2019 and 2024, dengue virus serotype data for 495 and 193 samples from North and South Goa respectively, show 58.57% of the samples to be DENV 2 serotype, followed by DENV 1 (21.51%), DENV 3 (17.87%), and DENV 4 (2.03%). There were no mixed infections of DENV serotypes detected.****”*** (line no. 333-336)

We acknowledge that the relatively small number of samples, particularly when stratified by district and year, may have limited our ability to detect mixed infections. The possibility that mixed infections were missed cannot be ruled out, and this has been noted as a limitation. We have clarified the sample size, testing method, and this limitation in the revised manuscript to improve transparency

*“The serotypes were tested on a limited sample, which may not accurately reflect the actual distribution of serotypes in the state, and the presence of mixed serotype infections couldn’t be captured.”* (line no. 652-654)

4. The introduction mentions underreporting of dengue but doesn’t explain how this might affect the study’s results or modeling. It would help to add a clear statement about how using underreported passive data can bias the findings and affect spatial and forecasting accuracy.

**Response:** We appreciate and agree with this suggestion. The revised manuscript has included this limitation to clearly explain how reliance on underreported passive surveillance data may influence the study findings. This clarification has been added to help readers interpret the results appropriately and understand the scope and limitations of the modelling approach.

***“***The data rely only on passive surveillance, which is prone to substantial under-reporting, and many individuals with mild or asymptomatic conditions do not seek medical attention. The woeful underreporting of dengue cases in India has also been highlighted in earlier studies [10]. This could lead to underestimation of absolute dengue burden in the state and may affect the precision of risk estimates and forecasting outputs, particularly in areas with variable healthcare access or diagnostic practices. However, the current analysis was based on routine surveillance data that are consistent throughout the study period; therefore, the spatial patterns and relative comparisons across locations and time remain informative.***”*** (line no. 637-644)

5. The Local Moran’s I and hotspot analyses in the study are performed using raw dengue case counts. Could this approach lead to misclassification of high-population Primary Health Centers (PHCs) as persistent hotspots, rather than truly high transmission areas? It is recommended to use normalized measures such as standardized incidence ratios (SIR), empirical Bayes rates, or expected case counts based on Poisson or negative binomial models for hotspot detection.

**Response:** We acknowledge the reviewer’s concern and agree that population-normalized measures (e.g., SIR or empirical Bayes rates) are preferable for risk-based inference. However, reliable year-wise, health centre-level catchment population data were not consistently available. Under these constraints, deriving expected counts or standardized rates could introduce additional uncertainty.

The use of raw dengue case counts is therefore justified in this study, as the primary objective was to identify persistent spatial concentrations of dengue burden and service-load pressure at the PHC level rather than per-capita transmission risk. Local Moran’s I remains statistically valid for exploratory clustering when applied uniformly across comparable spatial units and interpreted as burden-based clustering. This interpretation has been explicitly clarified in the revised Methods, Results, and Limitations sections.

6. The description of the Seasonal Mann–Kendall (SMK) test is technically incorrect. The SMK test does not remove seasonal effects; instead, it evaluates monotonic trends while accounting for them by comparing values within identical seasonal blocks (e.g., month-by-month). Moreover, autocorrelation in dengue data violates SMK assumptions. Without correction, trend p-values may be inflated.

**Response:** We thank the reviewer for pointing out this important aspect of the SMK test. As suggested, we have applied a modified SMK test based on the Hamid and Rao (1998) variance correction approach, and this has deflated the p-values for a few of the health centres. The Supplementary material has been updated to reflect the new modified SMK test table and the methodology and results have also been modified accordingly to define the modified test (with reference) as well as describe the new results. As seen in the results, even though the results for some health centres have changed, the overall finding (strong trends in North Goa and non-significant negative trends in South Goa) still remains the same.

7. The manuscript describes the Seasonal Mann–Kendall (SMK) test as removing seasonal effects, but is this characterization accurate? It is more precise to state that the SMK test evaluates monotonic trends by accounting for seasonality through separate analyses within each seasonal block (e.g., month-by-month). The test does not remove seasonal effects but controls for them by comparing data within these blocks. Additionally, because dengue data often exhibit autocorrelation, it would be beneficial to address this, as it can inflate significance levels and affect the validity of trend p-values.

**Response:** We thank the reviewer for pointing out this error in the description of the SMK test. We have corrected this description and have clearly stated that the SMK test “accounts” for seasonal effects by stratifying data into identical seasonal blocks.

8. The abstract reports a value of “≈ 3.94,” but it is unclear what this number represents. Is it a test statistic, Sen’s slope estimate, Z-score, or a metric derived from ArcGIS? Each of these has different implications and units. Please clarify the nature and units of this “≈ 3.94” value to help readers correctly interpret the direction and magnitude of the reported trend. Providing this information will enhance clarity and prevent misunderstanding of the results.

**Response:** The value reported is of the standardized Z statistic for the Mann-Kendall test. This has been stated more clearly both in the abstract and in the manuscript text.

9. Potential changes in PHC boundaries or administrative units were not addressed over a year. Such boundary changes can produce artificial spatial patterns and should be considered or explicitly stated.

**Response:** We thank the reviewer for highlighting this important consideration. During the study period, no officially notified major changes in PHC boundaries were reported by the district health authorities. However, we acknowledge that minor administrative adjustments and variations in service catchment areas may have occurred and could influence fine-scale spatial patterns.

To ensure transparency, we have explicitly stated this in the methodology and as a limitation in the revised manuscript, noting that the analysis assumes temporal stability of health center boundaries. All spatial analyses were conducted using a consistent health center boundary layer across all years to maintain methodological comparability. We clarify that the identified clusters should therefore be interpreted as indicative of persistent dengue burden patterns, rather than precise boundary-dependent risk estimates.

10. The study does not mention whether there were changes in PHC boundaries or administrative units over the study period. Could such boundary changes influence the spatial patterns observed? It would be beneficial to consider or explicitly state whether any changes in PHC or administrative boundaries occurred during the study period, as these can create artificial spatial patterns or discontinuities in the data.

**Response: We thank the reviewer for this valuable observation. During the study period, no officially documented major changes in PHC administrative boundaries were reported by the district health authorities. However, we acknowledge that minor administrative adjustments or variations in PHC service catchment areas may have occurred.**

**To address this concern, we have explicitly stated in the revised manuscript that all spatial analyses were conducted using a consistent PHC boundary dataset across all years, and that the analysis assumes temporal stability of PHC boundaries due to the absence of time-varying boundary data. This assumption and its potential influence on fine-scale spatial patterns are now clearly discussed in the Methods and Limitations sections, ensuring transparent interpretation of the results.**

11. The forecasting model uses only 13 annual observations per Primary Health Center (PHC). Is this sample size sufficient for building robust forecasts over a 5-year horizon? Using only 13 observations may result in unstable forecasts and increase the risk of overfitting. It is important to justify the chosen forecasting approach given the limited data and to describe model validation methods such as cross-validation or out-of-sample testing. Ideally, longer time series or alternative modelling techniques that can handle short series should be considered to improve forecast reliability.

**Response:** We acknowledge the limited length of the PHC-level time series (2011–2023; n = 13). However, the forest-based forecasting tool in ArcGIS Pro is specifically designed for short, noisy, and non-linear time series and does not rely on parametric assumptions that require long temporal depth. The ensemble of 100 regression trees reduces overfitting through aggregation and leverages shared temporal structure across PHCs, thereby stabilizing individual forecasts.

Model validation was performed using out-of-sample testing, with 2024 data held out for validation. Forecast performance was evaluated using RMSE, and uncertainty was explicitly quantified using 95% prediction intervals. The five-year forecasts (2025–2029) are therefore intended as indicative planning projections, with uncertainty appropriately communicated.

This justification and interpretation have been clarified in the revised Methodology.

12. Figures 4 and 5 do not display the 95% prediction intervals, despite their mention in the Methods. This omission can mislead readers regarding forecast certainty. Include shaded 95% PIs around forecast lines to visualize uncertainty.

**Response:** Figure 5 (Now Figure 6) has been revised to explicitly visualize forecast uncertainty. For time-series plots, 95% prediction intervals are now displayed as shaded bands around forecast lines, enabling clear interpretation of uncertainty.

13. Figures 4 and 5 do not display the 95% prediction intervals (PIs), although these intervals are mentioned in the Methods. Could this omission affect readers’ understanding of forecast uncertainty? Including shaded 95% prediction intervals in the forecast figures would improve transparency and help readers better interpret the uncertainty associated with predictions. This visual addition enhances the clarity and credibility of the forecasting results.

**Response:** Figure 4 (Now Figure 5) has been retained as a point-forecast map, and the caption has been revised to explicitly state that forecast uncertainty is quantified using 95% prediction intervals reported in the forecasting results. This clarification ensures transparent interpretation of spatial forecasts without altering the map design.

14. The forecasting model uses 2–3 year lags, while the Distributed Lag Non-Linear Model (DLNM) applies monthly lags (0–3 months). Could the manuscript more clearly distinguish these differing lag structures? It would improve clarity to explicitly state that the forecasting model incorporates multi-year lags to capture long-term trends, whereas the DLNM focuses on short-term monthly lag effects. Additionally, specifying that the rainfall thresholds (630 mm for North Goa, 607 mm for South Goa) represent monthly cumulative totals relevant to the monthly analysis would help readers properly interpret these values.

**Response**: We have clarified the distinction between the two lag structures in the revised manuscript. Specifically, we now explicitly state that the forest-based forecasting model incorporates multi-year (2 3 year) lags to capture long-term temporal dependence and inter-annual trends, whereas the DLNM is designed to quantify short-term associations using monthly lags (0 6 months) between rainfall and dengue incidence. We have also clarified that the rainfall thresholds (630 mm for North Goa and 607 mm for South Goa) represent monthly cumulative rainfall totals, consistent with the monthly resolution of the DLNM analysis.

The clarification distinguishing multi-year lags in the forecasting model from monthly lags in the DLNM has been added to the Methods section under ‘Time-series Forecasting’ and ‘Distributed Lag Non-Linear Model’. Clarification on monthly cumulative rainfall thresholds has been added at the first mention of rainfall thresholds in the Methods/Results section.

15. The rainfall thresholds are currently presented as a range (630–607 mm). Could expressing them this way lead to misunderstandings about their meaning? It is clearer and more precise to present the rainfall thresholds as distinct values for each district (630 [95% CI] mm for North Goa and 607 [95% CI] mm for South Goa). Presenting a range might mislead readers into interpreting these values as uncertainty intervals or generalized thresholds, whereas distinct values accurately reflect district-specific findings.

**Response:** We thank the reviewer for pointing out this ambiguity, which has now been corrected in the manuscript to clearly state the thresholds for North Goa and South Goa.

16. The manuscript uses a negative binomial generalized linear model (GLM) for count data. Was this choice justified by comparison to a Poisson model, which is commonly used for count data when variance equals the mean? Please consider including a justification for selecting the negative binomial model over a Poisson model. Typically, the negative binomial GLM is more appropriate when the data exhibit overdispersion i.e., when the variance exceeds the mean. Including diagnostic checks such as comparing residual deviance or likelihood ratio tests between Poisson and negative binomial models can support this choice and improve confidence in the analysis.

**Response:** In the revised manuscript, we have explicitly justified the use of the negative binomial (NB) GLM by demonstrating the presence of overdispersion in the dengue count data. Preliminary Poisson GLM fitting showed that the variance substantially exceeded the mean, and the residual deviance-to-degrees-of-freedom ratio was greater than one, indicating overdispersion. A likelihood ratio test comparing Poisson and negative binomial models further supported the NB model as providing a significantly better fit. These diagnostics are now clearly reported in the Methods section, thereby strengthening confidence in the modeling approach.

17. Variables such as temperature, rainfall, humidity, and pressure are often highly intercorrelated. Multicollinearity testing (VIF, condition index) should be reported since collinearity can inflate variance and distort incidence rate ratios.

**Response:** Both Spearman Rank Correlation test and VIF test were used for assessing the collinearity between predictor variables, with correlations greater than 0.8 and/or VIF greater than 10 indicating strong collinearity. The Methodology has been updated to clearly specify the criteria for checking the multi-collinearity in predictor variables. We have also updated the results section to provide a clear description of collinear variables and how variables were selected

18. The climatic variables used in the analysis such as temperature, rainfall, humidity, and pressure can be highly intercorrelated. Has multicollinearity among these predictors been assessed? It is advisable to test for multicollinearity using measures such as Variance Inflation Factor (VIF) or condition index. Reporting these assessments can help identify and mitigate potential collinearity issues, which can inflate the variance of coefficients and distort the interpretation of incidence rate ratios. Addressing multicollinearity improves model reliability and the robustness of epidemiological inferences.

**Response:** Both the Spearman Rank Correlation test and the VIF test were used for assessing the collinearity between predictor variables, with correlations greater than 0.8 and/or VIF greater than 10 indicating strong collinearity. The Methodology has been updated to clearly specify the criteria for checking the multicollinearity in predictor variables. We have also updated the results section to provide a clear description of collinear variables and how variables were selected.

19. The negative binomial generalized linear model (NB GLM) assumes independence among observations at different Primary Health Centers (PHCs). Given the spatial dependence typical in dengue transmission, is this assumption realistic? It is recommended to consider modeling approaches that account for spatial dependence, such as spatial generalized linear mixed models (GLMM), conditional autoregressive (CAR) models, or Besag-York-Mollié (BYM) models.

**Response:** We thank the reviewer for this important observation. We acknowledge that dengue transmission exhibits spatial dependence and that spatially explicit models (e.g., CAR/BYM) are well-suited for formal spatial risk estimation. In the present study, the negative binomial GLM was intentionally used as a baseline, non-spatial regression model to quantify associations between covariates and dengue counts while accounting for overdispersion.

Spatial dependence was explicitly addressed through separate spatial and spatio-temporal analyses, including Local Moran’s I, hotspot analysis, and space–time pattern mining to identify and characterize spatial clustering independently of the regression framework. This two-stage analytical strategy—non-spatial regression for covariate effects followed by spatial diagnostics for clustering—is a common and accepted approach in applied spatial epidemiology.

We now explicitly acknowledge in the revised manuscript that the NB GLM assumes conditional independence and that unmodeled spatial autocorrelation may remain. The use of spatial GLMMs or CAR/BYM models is therefore identified as an important direction for future work, particularly for inferential risk modelling.

This clarification has been added to the “Methods” to improve transparency.

20. The Distributed Lag Non-Linear Model (DLNM) is applied using monthly-aggregated data. Is this temporal resolution sufficient to accurately capture short-term lag effects critical to dengue transmission dynamics? It would be helpful to justify the choice of monthly aggregation for the DLNM analysis. If capturing short-term effects is essential, consider re-analyzing the data at a finer temporal resolution (e.g., weekly) if possible to improve the model's ability to detect and interpret short-term lagged associations. Providing this rationale strengthens the methodological rigor and supports interpretation of the results.

**Response:** We thank the reviewer for this comment. Monthly aggregation was used because dengue surveillance and meteorological data were consistently available and quality-assured at the monthly scale across the full study period, whereas finer (weekly) data were not uniformly available. Monthly resolution remains epidemiologically appropriate for dengue, as mosquito development, viral incubation, and reporting delays typically operate over weeks to months, which aligns with the 0–3 month lag window examined in the DLNM.

21. The manuscript describes land-use and Normalized Difference Built-up Index (NDBI) metrics but does not present statistical associations between these variables and dengue outcomes. Consider integrating land-use and NDBI metrics into the statistical models to explore their potential influence on dengue transmission. If the analysis was intended to be descriptive only, please clarify this explicitly.

**Response:** The analysis was intended for a better spatial visualization. It is now explicitly mentioned in the manuscript as “The health centre-wise dengue case distributions were overlaid on the land use change and NDBI maps for a better spatial visualization (association) of cases in the high built environments”.

22. The Root Mean Square Error (RMSE) values across Primary Health Centers (PHCs) range widely from 0.06 to 43.55, indicating variability in model performance. What could be causing this disparity? It would be helpful to analyze and discuss factors contributing to this variability, such as differences in data quality, sample size, or inherent variability in dengue incidence. Including additional model validation metrics like Mean Absolute Error (MAE) and R-squared (R²) would provide a more comprehensive assessment of model performance and help interpret the results more clearly.

**Response:** The wide range of RMSE values primarily reflects heterogeneity in dengue incidence dynamics across PHCs, including differences in baseline case volumes, inter-annual variability, and outbreak intensity. PHCs with consistently low counts tend to show small absolute errors, whereas PHCs experiencing episodic outbreaks or high variability exhibit larger RMSE values. Additional contributing factors include differences in data completeness and signal-to-noise ratios at the PHC level.

To improve interpretability, we have expanded the discussion to explicitly acknowledge this heterogeneity and its implications for forecast accuracy. We also clarify that RMSE was selected as the primary metric because it penalizes larger errors relevant for outbreak-prone PHCs.

23. The manuscript describes forest-based forecasting but does not discuss whether cross-validation or other measures to prevent overfitting were applied. Can the authors clarify how overfitting was addressed? It is recommended to include information on cross-validation procedures or other methods used to reduce overfitting in the forecasting models.

**Response**: We thank the reviewer for this important point. Overfitting was addressed through a combination of ensemble learning, out-of-sample temporal validation, and model constraints inherent to the forest-based forecasting framework in ArcGIS Pro. Specifically, predictions were generated from an ensemble of regression trees, which reduces variance through aggregation. Model performance was evaluated using temporal hold-out validation, with data from 2011–2023 used for training and 2024 reserved for out-of-sample validation, thereby avoiding information leakage. Forecast uncertainty was further assessed using 95% prediction intervals, which provide an additional safeguard against overconfident predictions.

These details have been explicitly clarified in the revised manuscript.

24. The manuscript does not describe how missing climatic or dengue case data were handled. Could the authors clarify their approach? Please specify the methods used to address missing data, such as imputation techniques, interpolation, or exclusion criteria.

**Response:** We did not face any issues of missing data in our dataset. The PHC-wise data provided by the NVBDCP for the study period did not have any missing values. Additionally, since climatic data was directly extracted from the ERA-5 hourly rasters, it did not have any missing values. We have added a statement in the Methodology (Data Sources section) to specify this.

25. The incidence values reported lack information on the denominators, such as population sizes or sources. Could the authors specify this information for each PHC or year in terms per 000’s population? Including the population size or source data used to calculate incidence rates is essential for proper interpretation.

**Response:** As clarified in question 1 above, the last Census was conducted in 2011, and all the population statistics in the state are estimated based on Census 2011. To have a proper outlook of the dengue cases vs population, a new supplementary table 05 is added in the revised manuscript with the health centre wise estimated population, total dengue cases reported during 2011-2024, annual incidence of dengue in Goa state, and average annual dengue incidence at health centers, annual incidence of dengue in Goa state.

26. PHC-level dengue case count data likely contain many months with zero reported cases. Has the analysis considered this zero-inflation in the data? It is recommended to discuss whether zero-inflation modeling approaches or data smoothing techniques were employed to handle an excess of zero counts. Addressing zero-inflation can improve model fit and inference accuracy for count data with many zero observations.

**Response:** We thank the reviewer for this important observation. We acknowledge that PHC-level dengue surveillance data include periods with zero reported cases, particularly in low-incidence months. In the present study, explicit zero-inflated models were not applied, as exploratory analysis indicated that the negative binomial (NB) GLM adequately accommodated excess variability and zero counts without evidence of structural zeros dominating the data. The NB framework is commonly used in epidemiological applications where zero counts arise naturally from low transmission rather than from a separate zero-generating process. To improve transparency, we have explicitly acknowledged the presence of zero counts and the rationale for not applying zero-inflated models in the revised manuscript.

27. Adding a limitations section that briefly discusses key issues such as size – number of data point, potential autocorrelation, lack of population adjustment, and the impact of temporal resolution would greatly enhance readability and provide context for interpreting the results. Such acknowledgement helps readers assess the robustness and applicability of the findings.

**Response:** We acknowledge that identifying the limitations of the study is critical to provide context for interpretation for the reviewers. We have, in fact, included a detailed paragraph on the limitations of the analysis at the end of the Discussions. Additionally, as per the reviewer’s recommendations, we have appended this paragraph with other relevant limitations mentioned by the reviewer.

28. The manuscript mentions the use of a space–time cube for hotspot analysis. Could the authors specify whether the temporal aggregation is annual, monthly, or quarterly? Clearly stating the temporal resolution of the space–time cube is important because the detection and interpretation of emerging or consecutive hotspots are highly sensitive to the chosen time-step.

**Response:** The space–time cube in this study was constructed using annual temporal aggregation, consistent with the temporal resolution of the dengue case data used for long-term spatio-temporal pattern analysis. We have now explicitly stated the annual time-step in the “Methods” section. The same is mentioned in the section on Space-time Analysis of Dengue in Goa.

29. The manuscript uses ERA5 temperature data, which are provided in Kelvin, but presents results in Celsius without explanation. Have the authors clearly documented this conversion? Please state explicitly in the Methods section that the temperature data in Kelvin were converted to Celsius during preprocessing.

**Response: Temperature was converted from Kelvin to Celsius for better clarity. As suggested, the Methodology has been updated to specify this.**

30. Figure 7 presents land-use changes, but does it include quantitative values such as built-up area in square kilometers? To enhance interpretability, please include quantitative measures of change (e.g., built-up area in km²) within the figure legend or as annotations on the figure.

**Response:** The quantitative measures of change in built-up area (in km²) are added in the caption of the land use/land cover figure.

31. The forecasting model described in the manuscript does not provide a full list of predictor variables. Could the authors provide this information? Please explicitly list all predictor variables included in the forecasting model and briefly justify why each was selected.

**Response:** In the revised manuscript, we have explicitly listed all predictor variables used in the forest-based forecasting model and provided a brief justification for each. The predictors were selected to capture temporal dependence, recent dengue dynamics, and long-term trends, consistent with the design of the ArcGIS Pro forest-based forecasting framework. This clarification has been added to the Methods section to improve transparency and reproducibility.

32. The manuscript mentions using cluster validity indices for time-series clustering. Are the values of these indices reported? Please include the cluster validity index values (e.g., silhouette score, Davies-Bouldin index) used to determine the optimal number of clusters. Presenting these metrics supports the choice of cluster numbers and improves the transparency and rigor of the clustering analysis.

**Response:** We thank the reviewer for this comment. In the revised manuscript, we have explicitly reported the cluster validity metrics used to determine the optimal number of time-series clusters. The clustering solution was evaluated using internal validity indices generated by the ArcGIS Pro time-series clustering framework, including the silhouette coefficient and within-cluster compactness/separation measures. The selected number of clusters corresponded to the configuration that maximized cluster separation while maintaining internal homogeneity.

33. The manuscript mentions reducing the number of climatic variables but it does not describe the details of those reduction? Please provide a clear description of the variable reduction details.

**Response:** We understand that the variable reduction method may not have been described in sufficient detail. We have appended the methodology to clearly describe the variable selection method, as per the reviewer’s suggestions. In short, the full DLNM models were initially fitted with all 13 climatic predictors, after which the outputs were screened for statistical relevance and compared with multi-collinearity tests. Posterior summaries of the full model were examined to identify and eliminate variables that showed no evidence or association or were weakly informative across all lag periods. Then, among the variables with significant cross-basis effects, all pairs of collinear variables were identified, and the ones with lower effects were eliminated to get the final set of significant variables. This is now clearly mentioned in the methodology.

34. The manuscript reports pseudo-R² and other model fit metrics but does so without contextual interpretation. Could these metrics be better explained? Please contextualize the reported pseudo-R² and related fit statistics by explaining what these values imply about model strength and predictive accuracy.

**Response:** We thank the reviewer for this suggestion. In the revised manuscript, we have contextualized the reported pseudo-R² and related fit statistics. Specifically, the pseudo-R² (~0.59) indicates that the negative binomial model explains a substantial proportion of the variability in dengue case counts, which is considered moderate to strong model performance for overdispersed count data. We clarify that pseudo-R² values are not directly comparable to ordinary least squares R², but rather reflect relative improvement over a null model. Additional diagnostics, including observed vs. predicted trends and residual distributions, are used to support model adequacy and predictive reliability. These interpretations are now explicitly stated in the methods sections.

35. The typo “17.87.%” should be corrected to “17.87%” by removing the extra period after the number. (Lines 266–269).

**Response:** Correction made (line 337)

36. The manuscript makes statements characterizing climate–disease associations as strong or weak. Are these statements supported by quantitative metrics? Please ensure that assertions about the strength of associations are accompanied by appropriate correlation coefficients (e.g., Pearson’s r, Spearman’s rho) or other relevant statistical measures.

**Response:** The study analyzed the climate disease associations using several different methods. First, a simple correlation test was applied, and the results were reported in Supplementary Table 7. As significant correlations were not identified, we have not specified the values in the main text. Secondly, we used a DLNM to assess lagged effects of all the climatic variables on dengue cases. Finally, self-exciting threshold models were used to identify variable thresholds. We have appended the manuscript to specify the quantitative metrics wherever needed.

37. The limitations don’t clearly mention the small sample size of serotype data and how that limits detecting changes over time or in small areas. The authors should also discuss how not using multiple data sources and model weaknesses might skew the results or cluster detection.

**Response:** The revised manuscript has included limitations on small sample size of serotype data.

*“The serotypes were tested on a limited sample, which may not accurately reflect the actual distribution of serotypes in the state, and the presence of mixed serotype infections couldn’t be captured.” (line 654-666)*

As mentioned in comment 02, data required to apply expansion factors from active surveillance, capture-recapture, or model-based corrections were not available for Goa. Therefore, we were unable to adjust for the underreporting, and this has been acknowledged as a limitation of the study.

*“The gross underreporting of dengue cases in India has also been highlighted in earlier studies [10]. This could lead to underestimation of absolute dengue burden in the state and may affect the precision of risk estimates and forecasting outputs, particularly in areas with variable healthcare access or diagnostic practices. However, the current analysis was based on routine surveillance data that are consistent throughout the study period; therefore, the spatial patterns and relative comparisons across locations and time remain informative.” (line 638-644)*

In the present study, the different methods—forest-based forecasting, climate-driven DLNM analysis, and hotspot detection were applied complementarily, each addressing distinct dimensions of dengue dynamics (temporal forecasting, climate sensitivity, and spatial clustering, respectively). This suggestion has been addressed in the Discussion section, where we now explicitly acknowledge methodological differences across forecasting and spatial models and identify integrated multi-model synthesis as an important direction for future research.

38. General suggestion is to carefully compare and combine results from different dengue forecasting methods like forest density, climate models, and spatio-temporal analyses. Since these methods use different data and approaches, their results may not always match; for example, hotspots found by the forest density model may differ from others as presented because of so many confounders or unknown factors. Putting all the data and results into one combined dataframe and analyzing them together using a suitable method will give a clearer and more accurate picture of dengue risk, author may consider this, if possible.

**Response**: We thank the reviewer for this valuable and forward-looking suggestion. We agree that integrating outputs from multiple forecasting and analytical frameworks can provide a more comprehensive assessment of dengue risk. In the present study, the different methods—forest-based forecasting, climate-driven DLNM analysis, and hotspot detection were applied complementarily, each addressing distinct dimensions of dengue dynamics (temporal forecasting, climate sensitivity, and spatial clustering, respectively). This suggestion has been addressed in the Discussion section, where we now explicitly acknowledge methodological differences across forecasting and spatial models and identify integrated multi-model synthesis as an important direction for future research.
