# Supplementary material for: Dengue transmission dynamics in an urban setting in western India
Source: PLoS Negl Trop Dis. 2026 Mar 23;20(3):e0013636. doi: 10.1371/journal.pntd.0013636 (PMC13052988; doi:10.1371/journal.pntd.0013636)
Supplement: Attachment — Submitted filename: PNTD-D-25-01818 comments.docx [file pntd.0013636.s015.docx]

**PNTD-D-25-01818**

Dengue transmission dynamics in an urban setting in western India by K Balasubramani et al.

**Reviewer comments**

Dengue is emerging in many parts of India, and there is a need to assess its risk using modern tools, including time-series analysis, for early forecasting and timely response. This study is therefore very relevant and addresses these needs in urban centres, both in the coastal zones in western India and elsewhere in the countries with similar ecologies.

This manuscript needs improvement for better readability. I have listed many suggestions below.

**Abstract**

On line 31: Replace “is critical’ with “was undertaken”

Line 35: Change “surveillance data” to “passive surveillance data routinely collected from 34 health facilities.”

Line 37: Change “clusters of dengue” to “clusters of dengue cases”

Line 43: Change “serotype” to “dengue virus serotype”—mention which other serotypes were also reported.

Line 44: Modify this sentence to “The clusters of dengue cases were predominantly observed in North Goa— the SMK test showed a significant positive trend for dengue cases in almost all the [give number] health facilities in the North Goa district.

Line 46: Change “show” to “showed”

Line 47: Change to “clusters in the PHCs Candolim, .....”

Line 51: Change “rainfall threshold of 630-607” to “total annual rainfall of 607 to 630 mm.”

Line 57: “early warning for dengue in Goa”

**Introduction**

Lines 87-88: Update the names of the states reporting the most dengue cases and deaths in India according to the data available from the NCVBDC website from <https://ncvbdc.mohfw.gov.in/index4.php?lang=1&level=0&linkid=431&lid=3715>

Lines 98-99: Instead of reporting cases in 2019 in Goa, update the dengue case numbers in the last 2-3 years in Goa (e.g. to approximately 600 cases and 3 deaths in 2024, or quote updated statistics from the Goa state sources).

Line 101: Cite more recent estimates of the economic burden of dengue (e.g. 2016 by Shepard et al. (Lancet Infect Dis. 2016 Aug;16(8):935-41. doi: 10.1016/S1473-3099(16)00146-8).

**Methodology**

Line 125-129: How many health facilities (PHCs, UHCs, CHCs) are there in total, and did the collected data on dengue cases cover all of them?

Line 127-128: Replace “Age sex wise data” with “data by age and sex”. Is NVBDCP still the correct acronym?

Lines 130-131: Clarify for which years the data were obtained/analysed (note: Reference # 16 was published in 2020).

Lines 133-134: The meteorological data source is from the year 2020 and the study period was 2011–2024, so how was it possible that the meteorological variables were computed for the period beyond 2020 when no data were obtained?

Line 139: Put “S1 and S2 Tables” in brackets.

Line 140: Use lowercase text in the subtitle.

Line 151: Use lowercase text in the subtitle.

Line 152: It is better to state “The dengue data by health facilities or health centres” because data were collected from the UHCs and CHCs as well. Later, you also mentioned PHCs only (e.g., lines 154, 157, 159, and so on).

Line 153: “location (Lat, Long)” to “geographical coordinates”.

Line 198: Use lowercase text in the subtitle.

Line 217: Use lowercase text in the subtitle.

Line 234: Use lowercase text in the subtitle. Here, you have rightly used the term “health centres”, so maintain uniformity.

**Results**

There is no mention of the number of dengue deaths in Goa. The national programme reported just 7 deaths in 2022-2024 (<https://ncvbdc.mohfw.gov.in/index4.php?lang=1&level=0&linkid=431&lid=3715>). Check the local health facility data to see if reporting of any deaths was missed.

Lines 238-240: In this sentence ending with “reported in North Goa”, insert “(**S5 Table**)” to inform the reader availability of detailed data in S5 Table. Delete “S5 Table” in line 248.

Line 240: By “>100” do you mean “>100 cases per health facility”?

Lines 240-244: This long sentence needs clarity– are you talking of centres located in two districts?

**Fig. 1A:** Split this single Figure 1A into three different figures since they convey different messages.

Line 255: Modify to “Mean monthly distribution ....”.

Lines 259- 260: The total number of cases was 4717 (see line 245 and S5 Table), so 61.49% cannot be just 1335 cases. Something is amiss here.

Line 260: Also, most cases in ≥ 15-year-olds could probably be because they constituted a majority in the total population. Without denominators (the total number of people in each age and sex group within the population being studied), you cannot calculate accurate rates or proportions.

Lines 262-264: In **Figure 1B**, what do the two different colours of data bars (blue and red) indicate?

Line 269-270: During 2020-2023, DENV-2 was the predominant serotype. In 2024, when only about 50 serotypes were reported, DENV-1 may have predominated. So, this does not indicate a rising trend for DENV-1.

Line 274-275: In this sentence, you mention **cumulative incidence per 100,000 persons**, but Table S5 presents dengue cases and not incidence. This is confusing and needs clarity.

Line 286: Use lowercase text in the subtitle.

Line 342: After the sentence “the most southern health centers would remain with < 20 cases”, clarify that the southernmost Canacona health centre might report a caseload of >40.

Line 372: Use lowercase text in the subtitle.

Line 377: It will be more logical to state that “The high incidence of dengue followed high rainfall.” Also, is it “temperature” or “mean monthly temperature”?

Fig. 6 and lines 394 and later: Replace “Precipitation” with “Rainfall” since the term precipitation may include snowfall, which does not happen in Goa.

Line 385: Do you mean to say that in some of the health centres, there was a positive correlation between rainfall and dengue cases? The pooling of health centre data to get a district trend (in Fig. 6) might have possibly lost a positive association. You may possibly check the correlation between the amount of rainfall in June-July (early rains) and the cases.

Line 401: Replace “total precipitation” with “The total annual rainfall...”

Line 407: What is LU/LC – land use/land use class? Try to minimise the use of abbreviations/acronyms to improve the readability of the text unless a term appears frequently such as more than 6 times in the manuscript).

**Discussion**

Line 425: Which year is correct – 2001 (see line 97) or 2011?

Line 430: More correctly, the upward trend was after 2020 and not 2012.

Line 433-434: Revisit this sentence in view of an earlier comment. If this inference is found true, discuss possible reasons, such as higher occupational exposures of adult males or something else.

Line 436-438: “Goa is also seeing the emergence of DENV-3 as a dominant serotype” is incorrect. The dominant serotype has remained DENV-2, although you may state that all four serotypes were cocirculating in all the years of data analysis (2019–2024; see Fig. 1B).

Line 444-445: Absence of mixed serotypes also indicates a low grade of transmission of DENV-1, 3 and 4.

Discuss the reporting of just a few dengue deaths in the last 3-4 years.

Line 508-516: In addition to serosurveys in the general population, would you also suggest conducting a study to assess the role of migrant labourers in carrying the dengue virus with different serotypes?

Line 518-519: Correct to total annual rainfall.

Line 529: Can you throw some light on how cases were diagnosed at the health centres – by NS1/RDT or other, and if non-availability of diagnostic facilities contributed to underreporting?

**References**

Format references according to the journal’s style of references.

Italicise species names.

Reference # 1: Change KUMARA to lowercase.

Reference # 13: correct the typo “Dengue casea” to “Dengue cases”

Reference # 14: correct the typo “019 to “2019”
